# Prenatal and neonatal housing conditions affect anxiety-like behavior in adulthood in rats and interact with brain-derived neurotrophic factor (BDNF) Val66Met to alter expression of BDNF and stress markers in the ventral hippocampus

**DOI:** 10.3389/fnmol.2026.1844602

**Published:** 2026-07-08

**Authors:** Maarten van den Buuse, Michelle Corrone, Emily J. Jaehne, Veronica Begni, Alessia Marchesin, Marco A. Riva

**Affiliations:** 1 School of Psychology and Public Health, La Trobe University, Melbourne, VIC, Australia; 2 Biological Psychiatry Unit, IRCCS Istituto Centro San Giovanni di Dio Fatebenefratelli, Brescia, Italy; 3 Department of Pharmacological and Biomolecular Sciences, University of Milan, Milan, Italy

**Keywords:** brain-derived neurotrophic factor, neonatal, prenatal, sex differences, stress

## Abstract

**Introduction:**

We investigated the interaction of the brain-derived neurotrophic factor (BDNF) gene variant, Val66Met, with the effect of prenatal/neonatal environmental conditions on anxiety-like behavior in adulthood in rats.

**Methods:**

In a genetic Val66Met rat model, we compared the effects of a high-enrichment/high-complexity early-life environment (HE) and a low-enrichment/low-complexity environment (LE).

**Results:**

Body weight was higher in both male and female HE rats compared to LE rats. Anxiety-like behavior on a plus maze or in an open field was enhanced in both male and female HE rats compared to LE rats. In contrast, following HE, only in females, adrenal weight was higher, and in the forced swim test, immobility was lower, and swimming was higher. Body weight and behavioral changes did not differ between BDNF genotypes. Fear conditioning and extinction were not affected. The effect of HE vs. LE condition on expression of BDNF, the antioxidant transcription factor, NRF2, and the glucocorticoid receptor, NR3C1, in the ventral hippocampus varied depending on genotype, and most of these changes were again only seen in females. There were no effects on the expression of the stress markers, SGK1 and FKBP5, or the mineralocorticoid receptor, NR3C2.

**Discussion:**

These results show persistent effects of early-life environment on anxiety-like behavior and gene expression of BDNF and stress markers in adulthood, with some effects showing sex- and Val66Met genotype specificity. These results may be important for our understanding of factors involved in the development of clinical anxiety and depression, and also have implications for animal welfare in the laboratory setting.

## Introduction

Anxiety disorders represent a broad group of mental illnesses manifesting in symptoms of worry, panic, and excessive fear (American Psychiatric Association, 2013). Prevalence of these disorders has been increasing in recent decades, with some estimates suggesting 5–10% of the global population is affected (Baxter et al., 2013; Javaid et al., 2023). Numerous causative factors have been implicated in anxiety disorders, including genetics, negative life events or early life stress, low socioeconomic status (SES), level of education, and substance use (Michael et al., 2007). Gender differences have been described, with a greater risk in women than in men (Altemus et al., 2014; Gater et al., 1998).

Early life stress or negative events during childhood, such as neglect, abuse, or maltreatment, have been suggested to significantly increase the risk of later development of anxiety disorders (Jonker et al., 2017; Smith and Pollak, 2020; Syed and Nemeroff, 2017). Animal models have been used to investigate the underlying mechanisms, using models of prenatal, neonatal, or adolescent stress (Buss et al., 2012; Creutzberg et al., 2021; Gobinath et al., 2014; Millstein and Holmes, 2007; Nishi, 2020; Weinstock, 2017). For example, rats that were allocated to a stressful early-life living environment where they received minimal nesting materials (Walker et al., 2017), showed higher anxiety-like behaviors in adulthood when compared to controls (Dalle Molle et al., 2012). The same study investigated this relationship in humans by utilizing questionnaires to assess anxiety and perception of parental warmth during childhood years. The results indicated that participants who experienced less parental warmth during childhood had an increased likelihood of having an anxiety disorder in adulthood (Dalle Molle et al., 2012). Conversely, many studies utilize environmental enrichment (EE), that is, psychosocial stimulation and engagement with the environment throughout early-life critical periods, which was suggested to be a protective factor against the development of anxiety-like disorders in adulthood (Dominguez-Oliva et al., 2025; Huttenrauch et al., 2016).

Genetic factors contribute a considerable amount to the risk and progression of anxiety disorders (Smoller et al., 2008). Brain-derived neurotrophic factor (BDNF) is found in large concentrations in the basal forebrain, cortex, and hippocampus (Bathina and Das, 2015; Notaras and van den Buuse, 2019). BDNF plays an important role in neuronal plasticity, survival, and growth, as well as learning, memory, and executive function (Binder and Scharfman, 2004; Corrone et al., 2026; Notaras and van den Buuse, 2020). The BDNF Val66Met polymorphism has been associated with an increased risk of developing anxiety disorders (Antolasic et al., 2024; Frustaci et al., 2008; Montag et al., 2010; Notaras et al., 2015), although results remain controversial (Notaras and van den Buuse, 2020). The Val66Met variant causes a reduction of activity-dependent release of neuronal BDNF, with an estimated 18% deficit in the heterozygous Val/Met genotype and a 29% deficiency in the Met/Met genotype in comparison to Val/Val (Chen et al., 2006). A mouse model of BDNF Val66Met showed increased anxiety-like behavior (Chen et al., 2006; Yu et al., 2012). However, more recently, a rat model of the Val66Met variant, Val68Met rats, showed no changes in anxiety-like behavior, although fear memory was reduced (Jaehne et al., 2022). These results suggest that any effect of Val66Met on anxiety-like behavior may depend on other factors, for example, environmental stress during development.

To investigate the long-term interaction of environmental stress and early-life environmental conditions with BDNF Val66Met, including brain molecular mechanisms involved, in the present study, we used a rat model of the Val66Met BDNF gene variant, Val68Met rats (Jaehne et al., 2022), and allocated them to either one of two early-life housing conditions. Consistent with suggestions to avoid the term “enrichment” (Ratuski and Weary, 2022), we defined these as either a low-stimulation/low-complexity environment (LE) with minimal cage space and nesting material, or a high-stimulation/high-complexity environment (HE) (Corrone et al., 2026). The latter condition included a larger cage and a frequently changed environment in the form of added toys, ladders, and other stimuli. These housing conditions were maintained during pregnancy (i.e., prenatal) and early postnatal development until weaning, after which the rats were kept under standard housing conditions. In adulthood, the animals were tested in a battery of behavioral tests (Jaehne et al., 2024) aimed at assessing anxiety and depression-like behaviors. We also investigated the expression of BDNF and a number of stress-related genes in the ventral hippocampus, which is involved in anxiety-like behavior (Adams et al., 2008; Bannerman et al., 2004; van der Veldt et al., 2025).

In line with previous research, it was predicted that high levels of environmental complexity would result in lower levels of anxiety-like behavior, while rats raised in a low complexity environment would demonstrate higher anxiety-like behaviors in adulthood, as this environment was predicted to be the more stressful condition. Additionally, it was expected that Met carriers would exhibit the greatest increase in anxiety-like behaviors and changes in expression of relevant stress markers due to their deficit in activity-dependent BDNF release.

## Methods

All experimental procedures were carried out in accordance with the National Health and Medical Research Council of Australia animal ethics guidelines and were approved by the La Trobe University Animal Experimentation Ethics Committee (Application AEC20006).

### Val68Met rats and housing conditions

Male (*n* = 20) and female (*n* = 33) Val68Met breeder rats with the heterozygous Val/Met genotype were imported from a breeding colony at the Australian Research Centre (Perth, Western Australia) to the La Trobe Animal Research and Teaching Facility (LARTF), and allowed to acclimate to the facility for at least 2 weeks. The founder breeders for this colony were generously provided by Drs. Caryl Sortwell and Timothy Collier from Michigan State University, MI, United States (Mercado et al., 2021).

Immediately following mating, female breeders were randomly housed in either a HE or LE environment (Corrone et al., 2026). The HE environment consisted of large ‘double-decker’ individually ventilated cages (IVC; Tecniplast, Italy), with the addition of abundant shredded paper nesting material and objects (wood blocks, chain, crinkle nest material, ladders, wooden balls, and sunflower seeds), which were altered weekly for environmental stimulation. The LE condition consisted of single-level open-top cages with less space, minimal shredded paper nesting material, and no added toys or seeds for stimulation (Supplementary Figure 1).

In total, 28 dams had litters which consisted of Val/Val, Val/Met, and Met/Met offspring in the expected approximate 1:2:1 ratio (Supplementary Table 1). There was no difference in dam age at mating, litter size, offspring genotype ratio, offspring sex ratio, or offspring age at weaning between the two housing conditions (Supplementary Table 1). The dams and their offspring were housed in LE or HE conditions from conception until weaning, after which all offspring were moved to IVC cages with standard, low-level enrichment. Offspring were housed in these standard enrichment IVC cages from weaning until the end of the study.

A total of 121 rats were used for behavioral analysis, with 7–12 rats of each genotype, sex, and rearing environment, creating 12 experimental groups (Table 1). No more than two of each genotype, sex, and housing condition per litter were allocated to each experimental group. Litter size from which offspring were selected did not differ between groups (Supplementary Table 2). At 2 weeks of age, all pups had ear clip tissue collected for genotyping so that they could be genotyped and assigned to groups based on their genotype at weaning at 3 weeks of age. Genotyping was done by Transnetyx (Cordova, TN, United States).

**Table 1:** Total number of rats per group by genotype, sex, and housing condition.

	Val/Val		Val/Met		Met/Met	
Housing condition	Males	Females	Males	Females	Males	Females
LE	10	10	11	11	10	10
HE	11	8	12	12	7	9

At 12 weeks, the rats were humanely euthanized for brain analysis. Following approved standard operating procedures, this included placing the animals in a 40.5 L euthanasia chamber, after which CO_2_ was infused into the chamber at a rate of approximately 12 L/min. The animals were closely observed and, when breathing had ceased, they were removed from the chamber and decapitated. The brain was rapidly removed from the skull and placed on an ice-cold plate. Because the ventral and dorsal hippocampus play a differential role in locomotor activity and anxiety (Adams et al., 2008; Bannerman et al., 2004), we dissected the ventral hippocampus separately from the dorsal hippocampus via a 50/50 split. Brain samples were frozen on dry ice and stored at −80 °C until shipment on dry ice to Milan, Italy, where analysis of gene expression was performed.

### Behavioral analyses

All behavioral tests occurred between 8 and 11 weeks of age (Jaehne et al., 2022; Jaehne et al., 2023). In the first week, the rats underwent an Elevated Plus Maze (EPM) test, followed by an open field test 3 days later. The next week, the rats underwent a 3-day fear conditioning analysis. Finally, in the third week, the rats were tested in a 2-day forced swim test protocol (Supplementary Figure 1). Behavioral testing was done between 9:00 a.m. and 2:00 p.m. All experimenters were blinded to genotypes and early housing conditions throughout the process of behavioral analyses.

#### Elevated plus maze (EPM)

The EPM was a plus-shaped platform elevated 50 cm from the ground, with four 50 cm long arms 10 cm in width. Two arms were enclosed with 50 cm high walls, and the other arms were open with no walls. In the center of the EPM was a 10 cm × 10 cm open square that allowed the rat to move between the arms. Each rat was positioned in the center of the plus maze and allowed to freely move for a duration of 5 min. A camera mounted above the maze captured the rat’s movement, and video tracking (Ethovision, Noldus, Netherlands) was used to analyze the time spent in each arm of the maze and the number of open and closed arms visited. While inquisitive of novel areas, due to their instinctive nature to avoid open areas, rats tend to avoid visiting the open arms of the maze (Pellow et al., 1985; Wall and Messier, 2001). Therefore, more time in the open arms and more open arm visits are measures postulated to demonstrate decreased anxiety-like behavior.

#### Open field

Rats were placed into an open enclosure approximately 100 × 100 cm in size with 50 cm high walls with no ceiling, and left to explore for 10 min. A camera mounted above the apparatus captured the rat’s movements, and video tracking (Ethovision, Noldus, Netherlands) was used to analyze behavior, including the total distance travelled and time spent in a 50 × 50 cm square center zone. Activity in the center zone of the open field is commonly used as a measure of anxiety-like behavior in rodents, and less time spent in the center zone is postulated to suggest greater anxiety-like behavior (Denenberg, 1969; Prut and Belzung, 2003).

#### Fear conditioning

Fear conditioning chambers (Med Associates, St. Albans, VT, United States) were utilized to measure rat fear learning, memory, and extinction (Jaehne et al., 2022; Maren, 2001). Two different context conditions were used evenly between rats. Context A comprised of no house light, aluminum walls, and stainless-steel rod floors wiped down with water and peppermint essence between trials. Context B had a house light, white acrylic walls with green tape patterns, and sawdust below the stainless-steel rod floor that was cleaned with water in between trials.

The first day measured fear learning acquisition. After a period of 3 min for habituation, over a total period of 11 min, the rats were administered three 30-s 80 dB Sound Pressure Level (SPL) tones (conditioned stimulus, CS), followed by a 1-s 0.7 mA foot shock occurring from the grid floors (unconditioned stimulus, US). Each administration of the CS and US pairing was followed by a 3 min no-stimulus interval. The second day assessed fear memory and extinction learning. Animals were placed into fear chambers in the opposite context from day 1 for a habituation period of 3 min, followed by 40 CS tones (30 s each) without any foot shocks. These sessions occurred over 27 min, with 5 s in between the tones.

All animal movements and freezing were captured by automated near-infrared tracking software (Video Freeze, Med Associates), which calculated the percentage of time the rat was freezing during each 30 s CS tone presentation. Day 1 data were divided into the three CS/US pairing components, whereas day 2 was split into four averaged periods of 10 CS each for analysis, and responses in trials 1–10 were used as a measure of fear memory (Jaehne et al., 2022; Jaehne et al., 2023).

#### Forced swim test

Immobility in the forced swim test was introduced as a behavioral test for evaluating antidepressant drug activity (Slattery and Cryan, 2012). While it has consequently also been used as a measure of depressive-like behavior, where immobility behavior was thought to represent despair or lack of coping, more recently, it has been argued that immobility is a measure of a switch from active to passive behavior in the face of an acute stressor, mediated by cognitive processes underlying behavioral adaptation and survival (Molendijk and de Kloet, 2015).

The apparatus was a clear Perspex cylinder (20 cm W × 50 cm H) filled up to 30 cm with water at 23 ± 2 °C. The testing was completed over 2 days, with habituation carried out on the first day, which involved placing the rats into the apparatus for 10 min. On the second day, rats were placed back into the apparatus for 5 min, and their behavior was recorded via video camera for later analysis. Rats were continuously monitored by the experimenter during the testing, and immediately following testing were dried and placed on a warm mat before being returned to their home cages.

Video recordings were scored for immobility and climbing behaviors by two individual scorers using Kinoscope software (Kokras et al., 2017), with a strong correlation between both scorers’ data sets (*r* = 0.94, *p* ≤ 0.001). Immobility behavior was characterized by a lack of any active swimming movements, often reflecting floating or freezing behaviors. Climbing behavior is an escape-directed behavior that represents the opposite of immobility and is defined as the upward swimming movement of the forearms against the side of the apparatus (Slattery and Cryan, 2012).

### Gene expression analysis

### RNA extraction

Total RNA was extracted using PureZOL RNA isolation reagent (Bio-Rad Laboratories Cat #732–6,890) according to the manufacturer’s protocol. To prevent DNA contamination, samples were then treated with DNAse (Thermo Fisher Cat. EN0521) following the manufacturer’s protocol. RNA concentration was quantified using a NanoDrop spectrophotometer (Thermo Fisher).

The ventral hippocampus samples were analyzed for expression levels of BDNF, as well as BDNF IV, BDNF 3’UTR Long, BDNF VI, and BDNF IX. Total BDNF expression is under the control of several promoters (Notaras and van den Buuse, 2019; Aid et al., 2007). Of these, BDNF IV expression is associated with neuronal activity (Zheng et al., 2012) and contextual fear expression (Bach et al., 2023). BDNF 3’UTR Long has been shown to regulate BDNF expression in dendrites vs. soma (Zheng et al., 2012) and following chronic stress (Oh et al., 2019). BDNF VI is involved in activity-dependent BDNF expression and is particularly found in distal dendrites (Baj et al., 2011). BDNF VI expression is increased following prenatal stress (Neeley et al., 2011) and in temporal lobe epilepsy (Martinez-Levy et al., 2016). Finally, BDNF IX is the only coding exon of the BDNF gene, while the other exons (I–VIII) are non-coding and regulate tissue-specific expression. Exon IX contains the entire precursor protein sequence, but little is known about its specific regulation (Nair and Wong-Riley, 2016).

The samples were also analyzed for expression of a number of stress markers. Nuclear factor erythroid 2-related factor 2 (NRF2) is a transcription factor regulating cellular responses against toxic and oxidative stress (Bhandari et al., 2021; He et al., 2020). NRF2 has been shown to stimulate BDNF expression (Mendez-David et al., 2015; Yao et al., 2021), particularly exon IX. Serum/glucocorticoid-regulated kinase 1 (SGK1) plays an important role in cellular stress responses, and its expression is increased by early-life stress (Millette et al., 2025) and in depression (Dattilo et al., 2020). Hippocampus FK506-binding protein 5 (FKBP5) interacts with early-life stress to mediate anxiety-like behavior (Criado-Marrero et al., 2019). The effect of stress-induced elevated HPA axis activity involves two types of glucocorticoid receptors in the brain: the glucocorticoid receptor, encoded by the nuclear receptor subfamily 3 group C member 1 (NR3C1) gene, and the mineralocorticoid receptor, encoded by the NR3C2 gene (Mifsud and Reul, 2018). Early-life stress has been shown to lead to long-term epigenetic changes in NR3C1 and NR3C2 expression (Palma-Gudiel et al., 2015; Siller Wilks et al., 2024).

#### RT-PCR

In order to evaluate gene expression, real-time polymerase chain reaction (qRT-PCR) was performed (CFX384 Real-Time system, Bio-Rad Laboratories) using the iTaq Universal Probes One-Step kit (Bio-Rad Laboratories, Cat#1725141). Primers and probes for *β*-Actin, GAPDH, BDNF isoform IV, BDNF 3’UTR Long, SGK1, FKBP5, NR3C1, and NR3C2 were obtained from Life Technologies; primers and probes for BDNF and NRF2 were obtained from Eurofins (see Supplementary Table 6 for probe catalogue numbers and primer sequences).

Samples were run in triplicate using *β*-Actin and GAPDH as housekeeping genes. Thermal cycling consisted of 10 min of incubation at 95 °C to allow retrotranscription, followed by 5 min at 95 °C to allow TaqMan Polymerase to activate, after which 39 PCR cycles were performed. Each cycle consists of 10 s of heating at 95 °C, to denature dsDNA, followed by 30 s at 60 °C for annealing and extension.

### Data analysis

Statistical analyses of behavioral and gene expression data were conducted using IBM Statistical Package for the Social Sciences (SPSS) version 26 (IBM, Chicago, IL, United States), with graphs created on GraphPad Prism (version 9; GraphPad Software, San Diego, CA, United States). An *a priori* power analysis was conducted prior to the investigation to confirm a sufficient sample size (Cohen, 1988); however, the sample size used was also consistent with prior research (Jaehne et al., 2022). Data were screened for both univariate and multivariate outliers. Normality was checked using skewness and kurtosis standardized z-scores, the Shapiro–Wilk test, and the Kolmogorov–Smirnov test. Homogeneity of variance was assessed using Levene’s test for Equality of Variance.

For all analyses of variance (ANOVA) of behavioral data, between-subject factors were sex (male or female), genotype (Val/Val, Val/Met, or Met/Met), and housing condition (LE or HE). EPM data were analyzed using univariate (time in open arm or distance travelled) or repeated-measures (open vs. closed arm) ANOVA. Differences between groups in the open field were analyzed using repeated-measures ANOVA with time spent in the outer and center zone as the within-subjects factors. Fear conditioning data were analyzed using repeated-measures (CS period freezing during acquisition and extinction learning) ANOVA. Differences between groups on the forced swim test were analyzed using univariate ANOVA (time immobile or time climbing).

qRT-PCR data analysis was performed using the efficiency-corrected model method. Normalization was performed using the arithmetic mean of β-actin and GAPDH, which were individually verified to be stable across experimental groups. The amplification efficiencies of both target and housekeeping genes were taken into account (Pfaffl, 2001). Outliers in each experimental group were identified with SPSS and excluded from the analyses. Data are presented as fold change% compared to LE-Val/Val (set at 100%). Future studies should aim to verify the present findings by using geometric averaging of additional reference genes, as recommended as a strong normalization strategy (Vandesompele et al., 2002).

Statistical significance in all cases was assumed at a *p-*value < 0.05. A Bonferroni adjustment was used for all post-hoc analyses where appropriate. Effect size was determined by partial eta squared, where the magnitude of effect sizes (≥0.01 small, ≥0.06 medium, and ≥0.14 large) was defined using previously published guidelines (Cohen, 1988; Richardson, 2011).

## Results

### Body weight

**Figure 1:**
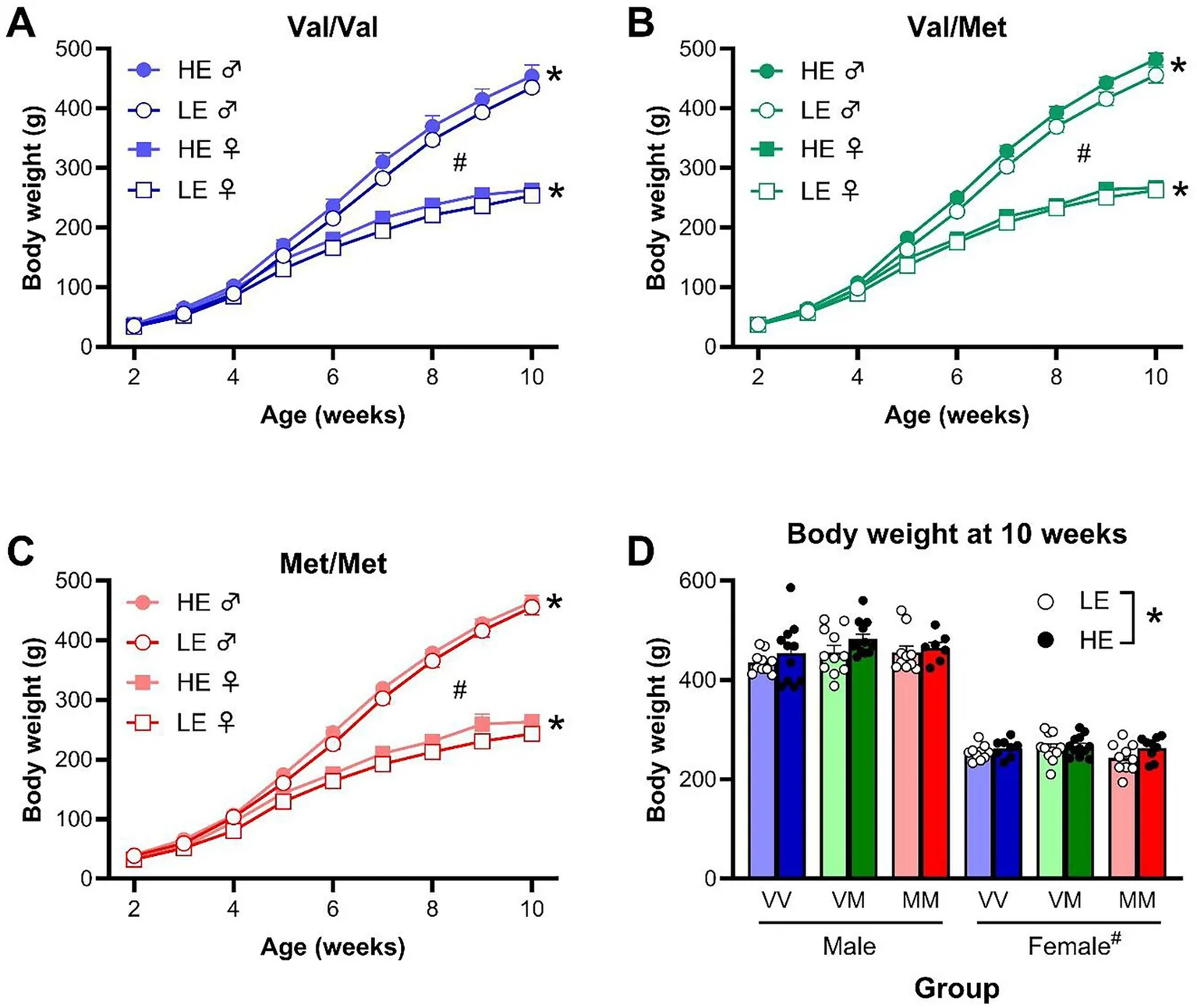
The effect of a high enrichment/high complexity early-life environment (HE) compared to a low enrichment/low complexity environment (LE) on body weight development from 2 to 10 weeks of age in male and female Val66Met rats of the Val/Val [Panel **(A)**], Val/Met [Panel **(B)**], and Met/Met [Panel **(C)**] genotype. Rats reared in the HE condition had significantly higher body weights than those reared in the LE condition, and male rats were significantly heavier than female rats, independent of genotype. [Panel **(D)**] shows body weight at 10 weeks of age. * *p* < 0.05 for difference between HE and LE across genotypes; # for difference between male and female rats. For the number of rats per group, see Table 1.

Rats reared in the HE condition were significantly heavier than those reared in the LE condition, independent of genotype. Repeated-measures ANOVA of offspring body weight from 2 to 10 weeks of age showed a significant main effect of housing condition [*F*(1,109) = 14.78, *p* < 0.001, *η_p_^2^* = 0.119] and a significant interaction between age and housing condition [*F*(8,872) = 4.87, *p* < 0.001, *η_p_^2^* = 0.043], with male and female rats reared in the HE condition heavier than those reared in LE (Figure 1). Further analysis of group differences at each age showed that rats reared in the HE condition were significantly heavier than LE rats at all ages, except at 2 weeks. There was also a main effect of age [*F*(8,872) = 7029.3, *p* < 0.001, *η_p_^2^* = 0.985], with body weight increasing over time as expected, as well as a main effect of sex [*F*(1,109) = 543.5, *p* < 0.001, *η_p_^2^* = 0.833] and an age × sex interaction [*F*(8,872) = 693.0, *p* < 0.001, *η_p_^2^* = 0.864] with males heavier and growing faster than females (Figure 1). Male rats were significantly heavier than female rats at all ages. There were no genotype differences in body weight at any age (Figure 1).

**Figure 2:**
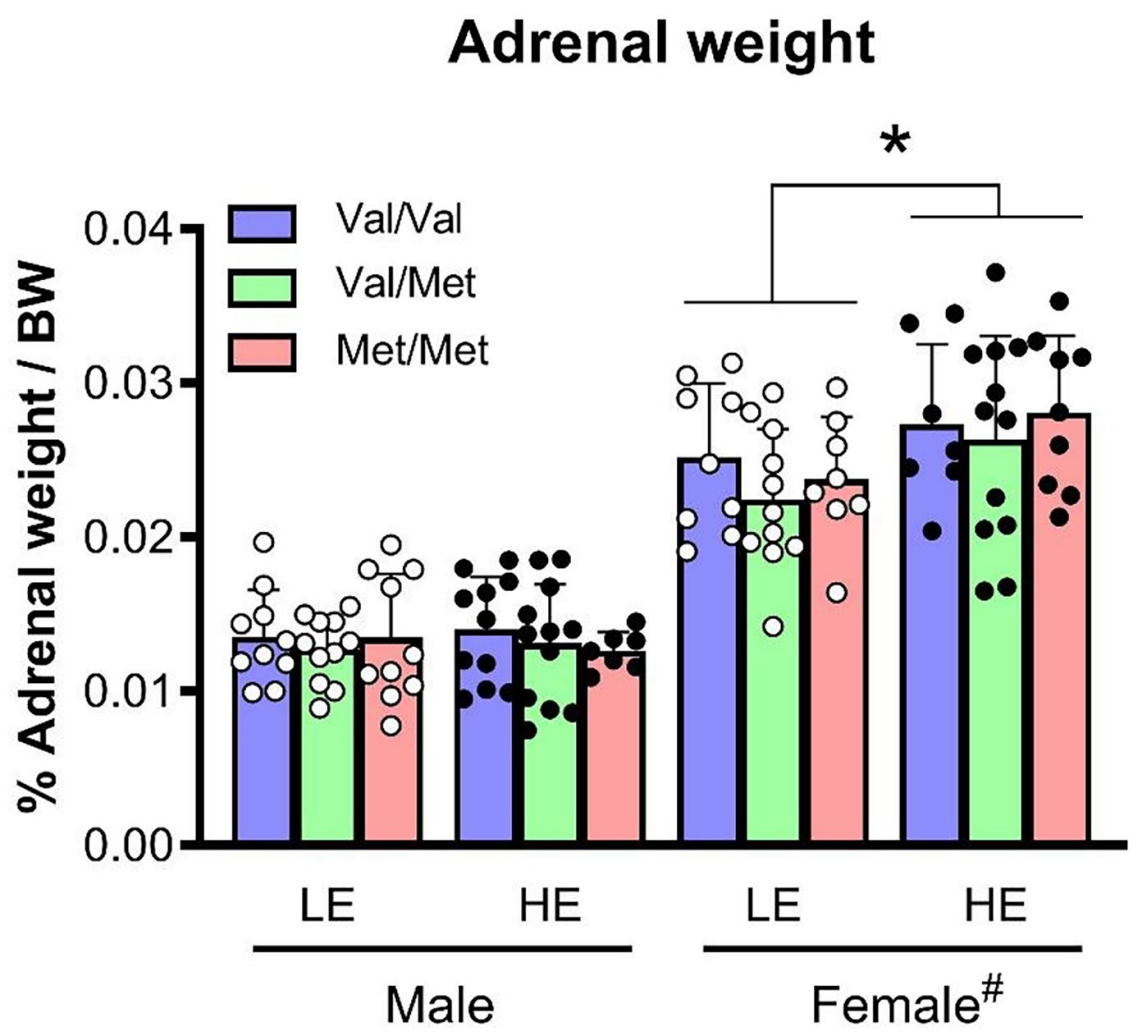
The effect of a high enrichment/high complexity early-life environment (HE) compared to a low enrichment/low complexity environment (LE) on adrenal weight/body weight (BW) ratio in male and female Val66Met rats of the Val/Val, Val/Met, and Met/Met genotype. Female rats reared in the HE condition had significantly heavier adrenals than females reared in the LE condition. Female rats had significantly heavier adrenals than male rats. These effects were independent of genotype. * *p* < 0.05 for difference between HE and LE across genotypes; # for difference between male and female rats. For the number of rats per group, see Table 1.

Adrenal weight corrected for body weight at the end of the study was significantly higher in females than in males [*F*(1,106) = 229.7, *p* < 0.001, *η_p_^2^* = 0.684]. There was also a trend for a sex × condition interaction [*F*(1,106) = 3.33, *p* = 0.071, *η_p_^2^* = 0.030]. Analysis of adrenal weights in males showed no significant differences between the groups. Female rats reared in the HE condition had significantly heavier adrenals than those reared in the LE condition [*F*(1,51) = 4.32, *p* < 0.05, *η_p_^2^* = 0.030], independent of genotype (Figure 2).

#### Elevated plus maze

**Figure 3:**
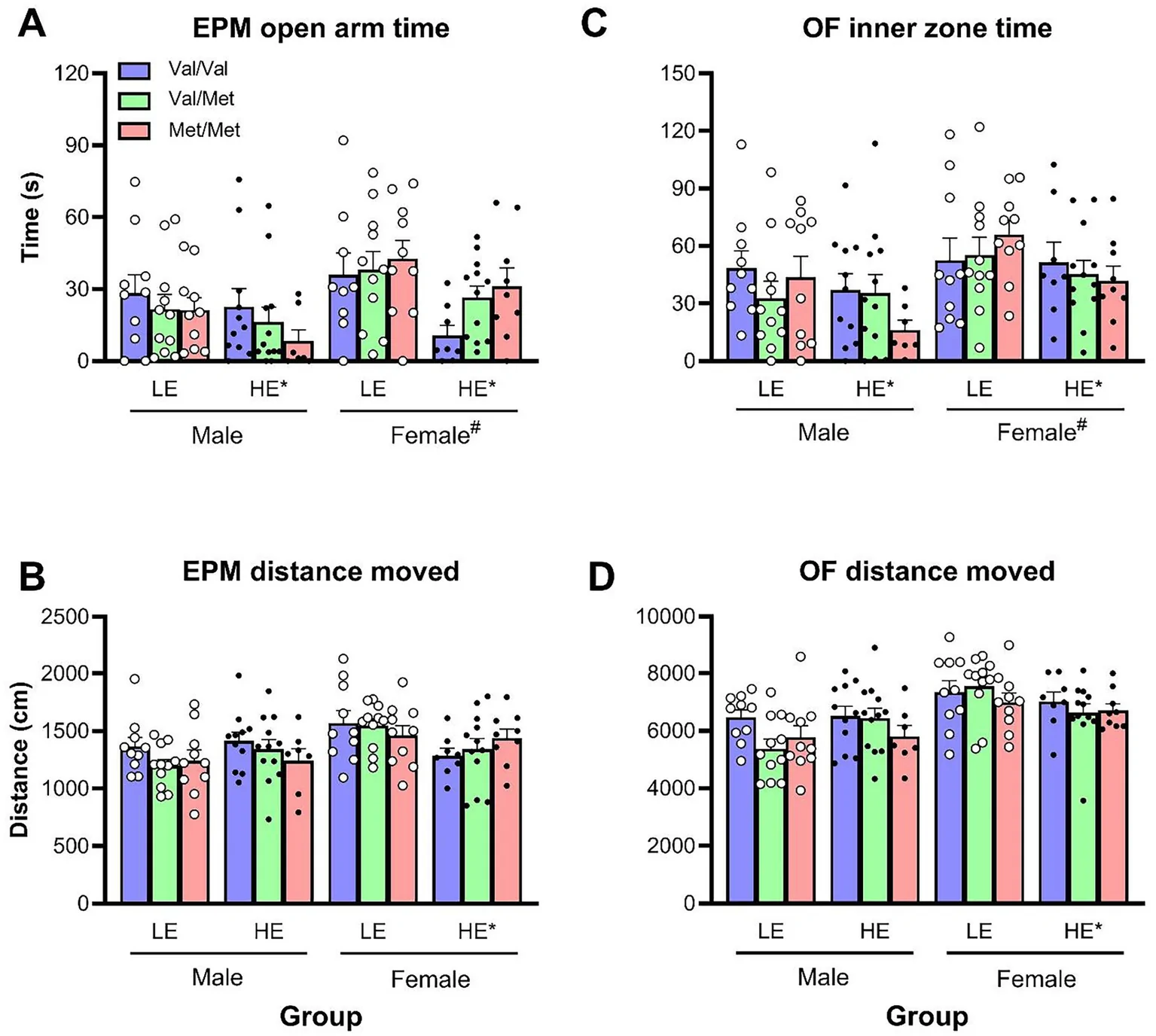
The effect of a high enrichment/high complexity early-life environment (HE) compared to a low enrichment/low complexity environment (LE) on open arm time on the elevated plus-maze (EPM) [Panel **(A)**], distance moved on the EPM [Panel **(B)**], inner zone time in the open field (OF) [Panel **(C)**] and distance moved in the OF [Panel **(D)**]. EPM open arm time was significantly lower in both HE males and HE females compared to their LE counterparts. EPM open arm time was significantly higher in females than in males [Panel **(A)**]. EPM distance moved was significantly lower in HE females compared to LE females, but not in males [Panel **(B)**]. OF inner zone time was significantly lower in both HE males and females compared to their LE counterparts [Panel **(C)**]. OF inner zone time was significantly higher in females than in males [Panel **(C)**]. OF distance moved was significantly lower in HE females compared to LE females, but not in males [Panel **(D)**]. These effects were independent of genotype. * *p* < 0.05 for difference between HE and LE across genotypes; # for difference between male and female rats. For the number of rats per group, see Table 1.

Rats reared in HE conditions appeared to show greater anxiety-like behavior, as suggested by less time on the plus maze open arms and, instead, more time on either the closed arms or the central square. There were no genotype differences in any of these effects (Figure 3). Repeated-measures ANOVA of time spent in open and closed arms with sex, genotype, and housing condition as between-group factors indicated a main effect for arm [*F*(1,109) = 1059.6, *p* < 0.001, *η_p_^2^* = 0.907], where rats spent more time in the closed arm compared to the open arm (Supplementary Table 3). There were interactions of arm with sex [*F*(1,109) = 4.25, *p* < 0.05, *η_p_^2^* = 0.038] and with housing condition [*F*(1,109) = 4.85, *p* < 0.05, *η_p_^2^* = 0.043]. Univariate ANOVA of time spent in the open arms showed a main effect of sex [*F*(1,109) = 9.07, *p* < 0.05, *η_p_^2^* = 0.077] and of housing condition [*F*(1,109) = 10.42, *p* < 0.05, *η_p_^2^* = 0.087] with female rats spending more time on the open arms than male rats and HE rats spending less time on the open arms than LE rats (Figure 3A). Analysis of time spent on the closed arms or in the central square showed no significant differences between the groups. However, the sum of time spent on the closed arms and in the central square was significantly higher in male rats than in female rats [*F*(1,109) = 9.04, *p* < 0.05, *η_p_^2^* = 0.077] and in HE rats compared to LE rats [*F*(1,109) = 10.42, *p* < 0.05, *η_p_^2^* = 0.087; Figure 3A; Supplementary Table 3].

Repeated-measures ANOVA of the number of entries in the open arms or closed arms only showed a main effect of arm [*F*(1,109) = 281.8, *p* < 0.001, *η_p_^2^* = 0.721] and no interactions. There was a main effect of sex [*F*(1,109) = 8.54, *p* < 0.05, *η_p_^2^* = 0.073] reflecting that female rats were more active and visited more arms on the elevated plus maze than male rats (Supplementary Table 3). This was also shown by analysis of total distance moved on the plus maze, which showed a main effect of sex [*F*(1,109) = 3166.6, *p* < 0.001, *η_p_^2^* = 0.967] with females moving a greater distance on the plus-maze than males (Figure 3B). However, ANOVA of distance moved also showed an interaction of sex and housing condition [*F*(1,109) = 5.88, *p* < 0.05, *η_p_^2^* = 0.051]. Further analysis of distance data split by sex showed that, independent of genotype, females in the HE condition travelled less distance on the plus maze than LE females [*F*(1,54) = 6.06, *p* < 0.05, *η_p_^2^* = 0.101] with no difference between HE and LE males (Figure 3B). Analysis of the data split by housing condition showed that, again independent of genotype, LE females travelled greater distances than LE males [*F*(1,56) = 1809.5, *p* < 0.001, *η_p_^2^* = 0.217], whereas there was no sex difference in distance moved by rats raised in the HE condition (Figure 3B).

#### Open field

Similar to the plus maze, rats reared in HE conditions appeared to show greater anxiety-like behavior in the open field, as suggested by HE rats spending less time in the inner zone of the open field than LE rats, independent of genotype (Figure 3). Repeated-measures ANOVA of time spent in the outer and inner zones of the open field with sex, genotype, and housing condition as between-group factors showed the expected main effect of zone [*F*(1,109) = 8904.1, *p* < 0.001, *η_p_^2^* = 0.988], with all rats spending more time in the outer zone compared to the inner zone (Supplementary Table 4). There were independent interactions of zone with sex [*F*(1,109) = 13.69, *p* < 0.001, *η_p_^2^* = 0.112] and with housing condition [*F*(1,109) = 4.49, *p* < 0.05, *η_p_^2^* = 0.040]. Univariate ANOVA of time spent in the inner zone of the open field revealed a main effect of sex [*F*(1,109) = 9.51, *p* < 0.05, *η_p_^2^* = 0.080] with female rats spending more time in the inner zone than male rats (Figure 3C). There was also a main effect of housing condition [*F*(1,109) = 5.04, *p* < 0.05, *η_p_^2^* = 0.044] with HE rats spending less time in the inner zone than LE rats (Figure 3C). Analysis of time spent in the outer zone (Supplementary Table 4) predictably showed the opposite trends with males spending more time in the outer zone than females [*F*(1,109) = 18.11, *p* < 0.001, *η_p_^2^* = 0.142] and a strong trend for HE rats to spend more time in the outer zone than LE rats [*F*(1,109) = 3.87, *p* = 0.052, *η_p_^2^* = 0.034].

Analysis of the number of entries from the outer zone to the inner zone of the open field revealed a main effect of sex [*F*(1,109) = 16.05, *p* < 0.001, *η_p_^2^* = 0.128] but no significant interactions, with females more active than males (Supplementary Table 4). Analysis of distance moved in the open field similarly revealed a main effect of sex [*F*(1,109) = 24.15, *p* < 0.001, *η_p_^2^* = 0.181] with females again more active than males (Figure 3D). Similar to distance moved on the plus-maze, analysis of distance moved in the open field also showed a sex × housing condition interaction [*F*(1,109) = 4.99, *p* = 0.027, *η_p_^2^* = 0.044]. Analysis of the data split by housing condition showed that LE female rats were more active than LE male rats [*F*(1,56) = 25.75, *p* < 0.001, *η_p_^2^* = 0.315], but there was no significant sex difference in distance moved in the open field between the HE groups (Figure 3D). Analysis of the data split by sex showed no effect of housing condition in males, with a trend for LE females to be more active than HE females [*F*(1,54) = 3.56, *p* = 0.064, *η_p_^2^* = 0.062]. There were no differences between the genotypes in any of these effects in the open field (Figure 3).

#### Fear conditioning

**Figure 4:**
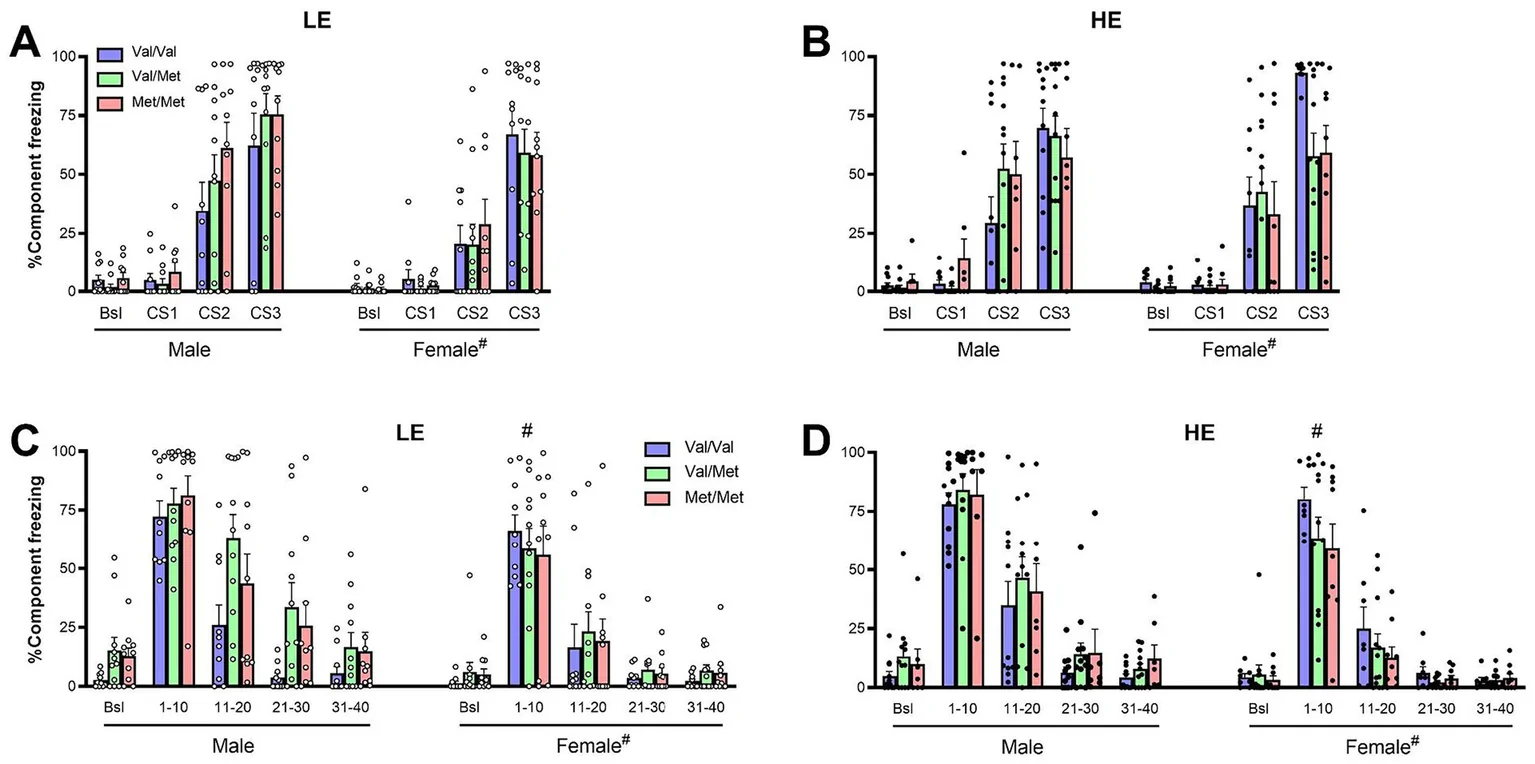
The effect of a high enrichment/high complexity early-life environment (HE) compared to a low enrichment/low complexity environment (LE) on acquisition of freezing [Panels **(A,B)**] and extinction learning [Panels **(C,D)**]. During acquisition, freezing significantly increased, reflecting fear learning [Panels **(A,B)**]. During fear extinction learning, freezing significantly decreased over repeated presentation of the CS in the absence of the US [Panels **(C,D)**]. Males showed higher freezing than females both during acquisition and extinction (# for difference between male and female rats, Supplementary Table 5). There were no differences (Supplementary Table 5) between the LE condition [Panels **(A,C)**] and the HE condition [Panels **(B,D)**]. For the number of rats per group, see Table 1.

There were no effects of HE or LE conditions on the acquisition of freezing or extinction learning (Figure 4). Repeated-measures ANOVA of fear acquisition showed a main effect for CS [*F*(2,218) = 216.9, *p* < 0.001, *η_p_^2^* = 0.666], reflecting that freezing increased over the three tones (Figures 4A,B). There was also an interaction of sex and CS [*F*(2,218) = 3.10, *p* < 0.05, *η_p_^2^* = 0.028], with males freezing more than females (Figures 4A,B). Additionally, there was a significant interaction between genotype and CS [*F*(4,218) = 2.89, *p* < 0.05, *η_p_^2^* = 0.050], suggesting subtle differences between the genotypes in the acquisition of freezing to the CS. However, there were no differences between the groups for CS3, suggesting that at the end of the session, all rats had similarly learned the association between CS and US (Figures 4A,B).

Analysis of fear extinction learning on Day 2 demonstrated a main effect of CS block [*F*(2.18, 237.6) = 365.9, *p* < 0.001, *η_p_^2^* = 0.770], showing the expected extinction of freezing over repeated presentation of the CS in the absence of the US. Analysis also indicated a significant interaction of CS block and sex [*F*(2.18, 237.6) = 5.53, *p* < 0.05, *η_p_^2^* = 0.048], accompanied by a significant main effect of sex [*F*(1,109) = 21.06, *p* < 0.001, *η_p_^2^* = 0.162], with males freezing more than females (Figures 4C,D; Supplementary Table 5). There were no significant effects or interactions of genotype or early housing condition. Using the first CS block as a measure of fear memory (Jaehne et al., 2022; Jaehne et al., 2023), ANOVA showed lower freezing in females than in males [*F*(1,109) = 10.09, *p* < 0.05, *η_p_^2^* = 0.085], but there were no effects of genotype or housing condition (Figures 4C,D; Supplementary Table 5).

#### Forced swim test

**Figure 5:**
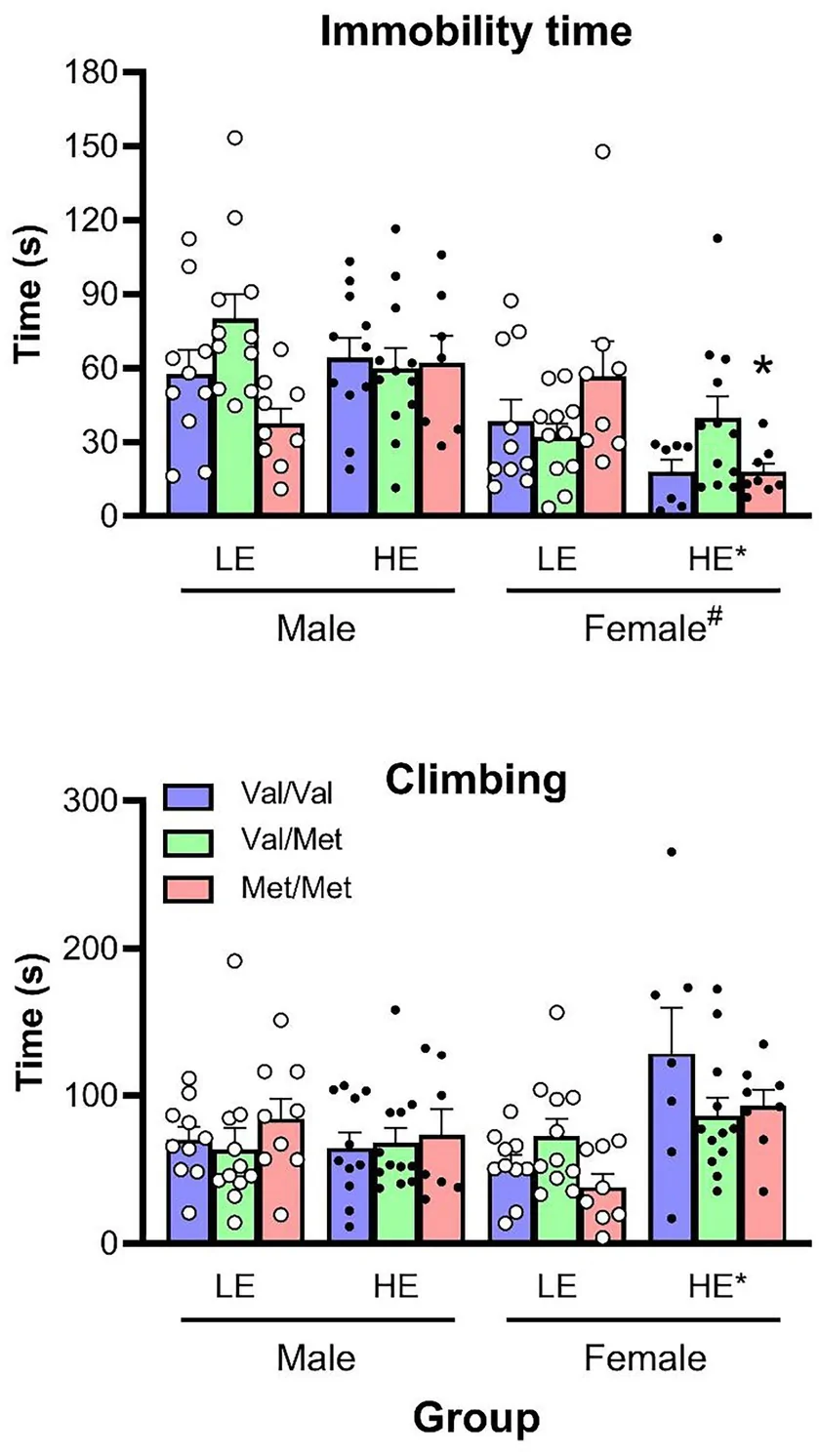
The effect of a high enrichment/high complexity early-life environment (HE) compared to a low enrichment/low complexity environment (LE) on immobility time (top panel) and climbing (bottom panel) in the forced swim test. Female HE rats showed less immobility and more climbing in the forced swim test than female LE rats, with no difference in males. Immobility was significantly lower in female HE Met/Met rats compared to LE Met/Met females, with no difference in Val/Val and Val/Met females. * *p* < 0.05 for the difference between HE and LE; # for the difference between male and female rats. For the number of rats per group, see Table 1.

Female HE rats showed less immobility and more climbing in the forced swim test than female LE rats, with no difference in males (Figure 5). Analysis of immobility time revealed a main effect of sex [*F*(1,104) = 26.4, *p* < 0.001, *η_p_^2^* = 0.202], with males showing greater immobility time than females (Figure 5). There was also an interaction of sex and housing condition [*F*(1,104) = 4.06, *p* < 0.05, *η_p_^2^* = 0.038], with further analysis by sex showing that female HE rats showed significantly less immobility than female LE rats [*F*(1,50) = 5.92, *p* < 0.05, *η_p_^2^* = 0.106] with no difference in males. This effect of housing condition in females was also dependent on genotype [*F*(2,50) = 4.02, *p* < 0.05, *η_p_^2^* = 0.138] with further analysis showing that immobility was significantly reduced in HE Met/Met rats compared to LE Met/Met rats [*F*(1,14) = 6.96, *p* < 0.05, *η_p_^2^* = 0.332] with no housing condition effect in Val/Val or Val/Met rats (Figure 5).

Analysis of climbing behavior showed both a main effect of housing condition [*F*(1,104) = 8.45, *p* < 0.05, *η_p_^2^* = 0.075] and an interaction of sex and housing condition [*F*(1,104) = 11.7, *p* < 0.001, *η_p_^2^* = 0.101] with further analysis by sex showing that, independent of genotype, female HE rats showed significantly more climbing than female LE rats [*F*(1,50) = 17.4, *p* < 0.001, *η_p_^2^* = 0.259] with no difference in males (Figure 5).

#### Gene expression analysis: BDNF

**Figure 6:**
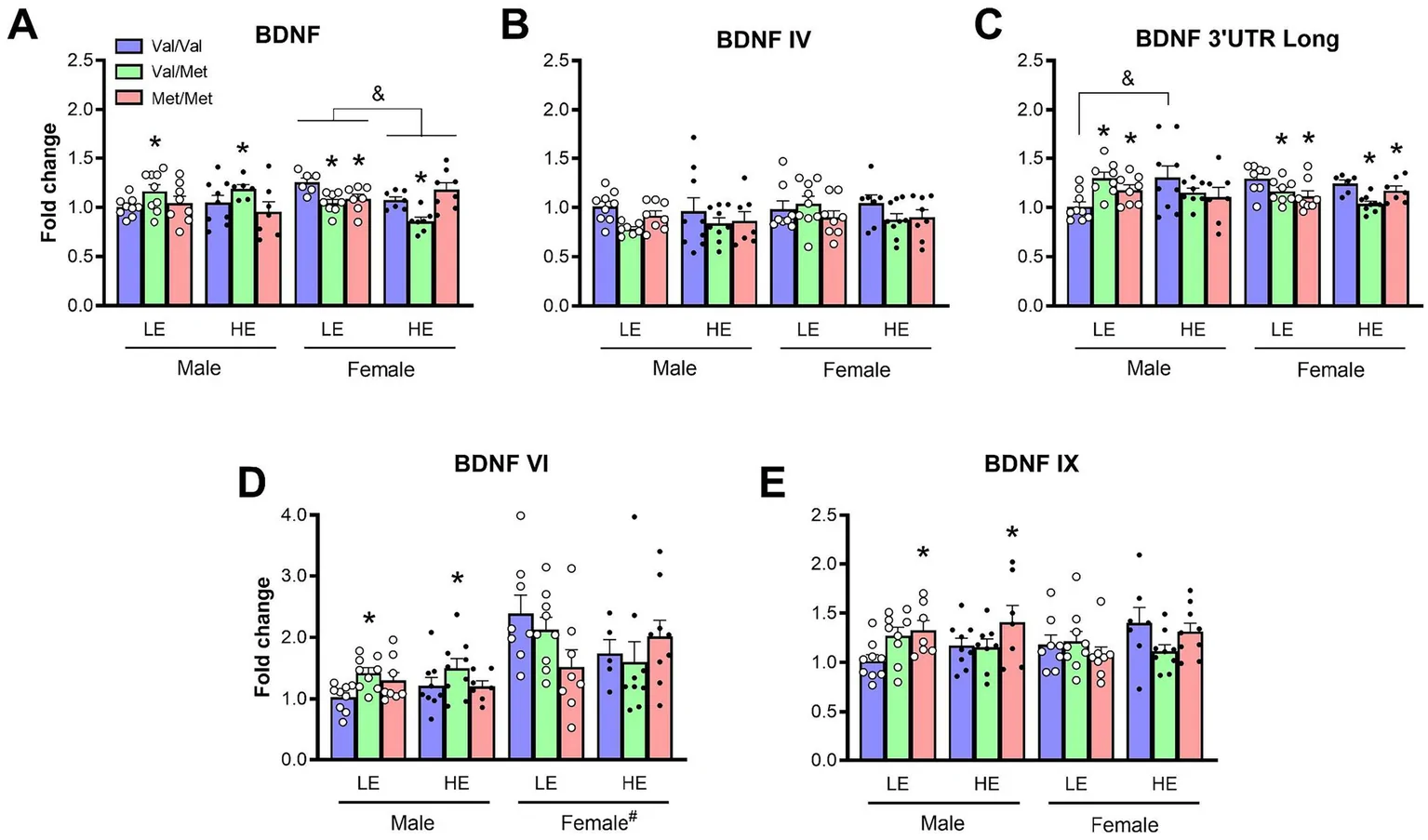
The effect of a high enrichment/high complexity early-life environment (HE) compared to a low enrichment/low complexity environment (LE) on expression of BDNF [Panel **(A)**], BDNF IV [Panel **(B)**], BDNF 3’UTR Long [Panel **(C)**], BDNF VI [Panel **(D)**], and BDNF IX [Panel **(E)**]. BDNF gene expression in males was significantly higher in Val/Met than both Val/Val and Met/Met, independent of environmental conditions [Panel **(A)**]. In females, BDNF expression was significantly lower in HE compared to LE rats [Panel **(A)**] and in Val/Met females compared to Val/Val females. BDNF expression was significantly lower in female Met/Met rats than in female Val/Val rats in the LE condition but not in the HE condition [Panel **(A)**]. There were no significant effects of sex, genotype, or early housing condition on BDNF IV expression [Panel **(B)**]. Expression of BDNF 3’UTR Long was significantly higher in male Val/Val rats in the HE condition than in the LE condition, but there were no differences in male Val/Met or Met/Met rats [Panel **(C)**]. In the LE condition, BDNF 3’UTR Long expression was significantly higher in Val/Met and Met/Met rats than in Val/Val males, but there was no difference between Val/Met and Met/Met rats in the LE condition or any genotype differences in the HE condition [Panel **(C)**]. In females, both Val/Met and Met/Met rats showed lower expression of BDNF 3’UTR Long than Val/Val females, with no effects of housing condition [Panel **(C)**]. BDNF VI expression was higher in females than in males. In males, Val/Met showed higher BDNF gene expression than Val/Val but not Met/Met [Panel **(D)**]. There were no effects of early housing condition or genotype on BDNF VI expression in females [Panel **(D)**]. BDNF IX expression in Met/Met was higher than in Val/Val but not in Val/Met, independent of housing condition, with no differences in females [Panel **(E)**]. * *p* < 0.05 for difference between genotypes; & *p* < 0.05 for difference between HE and LE; # *p <* 0.05 for difference between males and females. For the number of rats per group, see Table 1.

BDNF expression in the ventral hippocampus was significantly reduced in HE females compared to LE females, with no housing condition effect in males, and appeared to be inversely dependent on genotype between males and females (Figure 6A). Specifically, analysis of BDNF gene expression revealed a large genotype × sex interaction [*F*(2,79) = 11.95, *p* < 0.001, *η_p_^2^* = 0.232]. Further analysis split by sex of the animals showed a genotype effect in males [*F*(2,42) = 3.76, *p* = 0.031, *η_p_^2^* = 0.152], independent of housing condition, with Val/Met males showing higher BDNF gene expression than both Val/Val [*F*(1,29) = 6.39, *p* = 0.017, *η_p_^2^* = 0.181] and Met/Met males [*F*(1,26) = 5.69, *p* = 0.025, *η_p_^2^* = 0.180] but no difference between male Val/Val and Met/Met rats (Figure 6A). In contrast, in females, there were main effects of genotype [*F*(2,37) = 11.76, *p* < 0.001, *η_p_^2^* = 0.389] and housing condition [*F*(1,37) = 5.20, *p* = 0.028, *η_p_^2^* = 0.123] as well as a genotype × housing condition interaction [*F*(2,37) = 5.99, *p* = 0.006, *η_p_^2^* = 0.245]. BDNF expression was significantly lower in female HE rats compared to female LE rats (Figure 6A). Further analysis of the genotype differences showed that BDNF expression was significantly lower in Val/Met females than Val/Val females [*F*(1,24) = 32.2, *p* < 0.001, *η_p_^2^* = 0.565], irrespective of housing condition. In contrast, comparison of female Val/Val and Met/Met rats showed an interaction of genotype and housing condition [*F*(1,24) = 6.95, *p* = 0.014, *η_p_^2^* = 0.224] with BDNF expression significantly lower in female Met/Met rats than female Val/Val rats in the LE condition [*F*(1,11) = 6.61, *p* = 0.026, *η_p_^2^* = 0.375] but not in the HE condition (Figure 6A).

There were no significant effects of sex, genotype, or housing condition on BDNF IV expression (Figure 6B).

Similar to BDNF expression, analysis of BDNF 3’UTR Long suggested housing condition effects that were dependent on both genotype and sex (Figure 6C). ANOVA showed an interaction of housing condition, genotype, and sex [*F*(2,83) = 3.68, *p* = 0.029, *η_p_^2^* = 0.082] as well as interactions of housing condition and genotype [*F*(2,83) = 4.13, *p* = 0.019, *η_p_^2^* = 0.091] and of genotype and sex [*F*(2,83) = 3.42, *p* = 0.037, *η_p_^2^* = 0.076]. Analysis of the data split by sex revealed a housing condition × genotype interaction in males [*F*(2,43) = 4.95, *p* = 0.012, *η_p_^2^* = 0.187]. Regarding genotype-dependent condition effects, further analysis showed that expression of BDNF 3’UTR Long was significantly higher in male Val/Val rats in the HE condition than in the LE condition [*F*(1,16) = 5.30, *p* = 0.035, *η_p_^2^* = 0.249], but there were no effects of housing condition in male Val/Met or Met/Met rats (Figure 6C). Regarding housing condition-dependent genotype differences in males, in the LE condition BDNF 3’UTR Long expression was significantly higher in Val/Met [*F*(1,15) = 14.24, *p* = 0.002, *η_p_^2^* = 0.487] and Met/Met rats [*F*(1,15) = 5.55, *p* = 0.032, *η_p_^2^* = 0.270] than in Val/Val males but there was no difference between Val/Met and Met/Met rats in the LE condition or any genotype differences in the HE condition (Figure 6C).

In females, there was only a main effect of genotype [*F*(2,40) = 7.21, *p* = 0.002, *η_p_^2^* = 0.265], with both Val/Met [*F*(1,27) = 16.67, *p* < 0.001, *η_p_^2^* = 0.382] and Met/Met rats [*F*(1,25) = 5.79, *p* = 0.024, *η_p_^2^* = 0.188] showing lower expression of BDNF 3’UTR Long than Val/Val females but no difference between Val/Met and Met/Met females. There were also no effects of housing condition on expression of BDNF 3’UTR Long in females (Figure 6C).

Analysis of BDNF VI expression revealed a large main effect of sex [*F*(1,87) = 25.4, *p* < 0.001, *η_p_^2^* = 0.266], with females showing higher expression than males (Figure 6D). There was also an interaction of housing condition × genotype × sex [*F*(2,87) = 3.17, *p* = 0.047, *η_p_^2^* = 0.068], which was further analyzed by splitting the data by sex. This showed a genotype effect in males [*F*(2,45) = 4.54, *p* = 0.016, *η_p_^2^* = 0.168], independent of housing condition, with Val/Met males showing higher BDNF gene expression than Val/Val [*F*(1,32) = 8.40, *p* = 0.007, *η_p_^2^* = 0.208] but not Met/Met males and there was no difference between male Val/Val and Met/Met rats (Figure 6D). There were no effects of early housing condition or genotype on BDNF VI expression in females (Figure 6D).

Analysis of BDNF IX expression showed a large genotype × sex interaction [*F*(2,87) = 3.71, *p* = 0.028, *η_p_^2^* = 0.079], which was further analyzed by splitting the data by sex. This showed a genotype effect in males [*F*(2,43) = 4.02, *p* = 0.025, *η_p_^2^* = 0.158], independent of housing condition, with Met/Met males showing higher BDNF gene expression than Val/Val [*F*(1,28) = 7.32, *p* = 0.011, *η_p_^2^* = 0.207] but not Val/Met males and there was no difference between male Val/Val and Val/Met rats (Figure 6E). There were again no effects of early housing condition or genotype on BDNF IX expression in females (Figure 6E).

#### Gene expression analysis: stress-related factors

**Figure 7:**
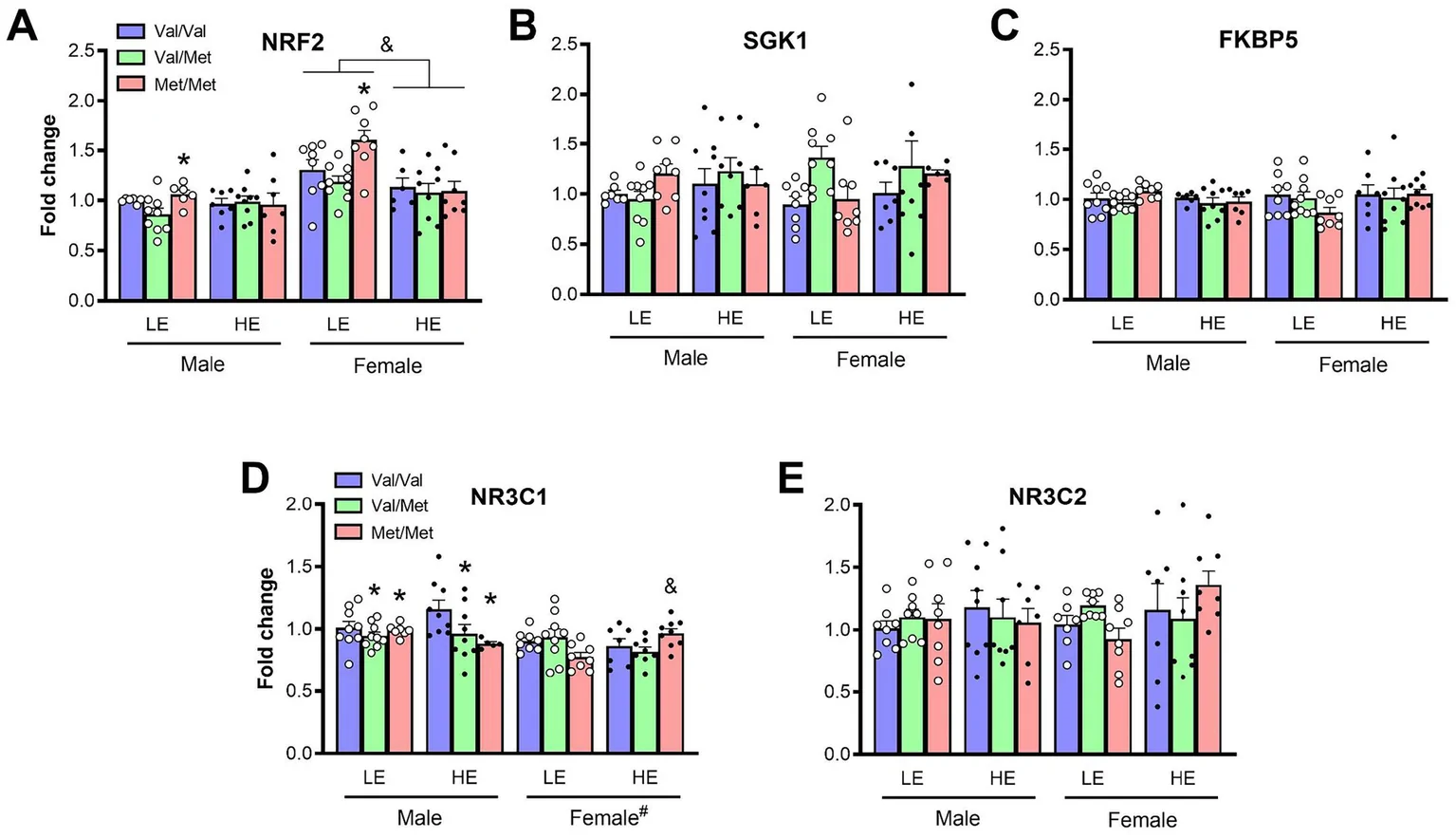
The effect of a high enrichment/high complexity early-life environment (HE) compared to a low enrichment/low complexity environment (LE) on expression of NRF2 [Panel **(A)**], SGK1 [Panel **(B)**], FKBP5 [Panel **(C)**], NR3C1 [Panel **(D)**], and NR3C2 [Panel **(E)**]. Expression of NRF2 was lower in females in the HE condition compared to the LE condition, independent of genotype, but no housing condition effect in males [Panel **(A)**]. NRF2 expression was higher in Met/Met rats compared to Val/Val and Val/Met rats in the LE condition but not the HE condition [Panel **(A)**]. There were no effects of genotype or housing condition and no sex differences in the expression of SGK1 [Panel **(B)**] or FKBP5 [Panel **(C)**]. NR3C1 expression was significantly lower in females than in males. In males, both Val/Met and Met/Met showed lower NR3C1 expression than Val/Val males [Panel **(D)**]. In females, NR3C1 expression was higher in Met/Met females in the HE condition than in the LE condition [Panel **(D)**]. There were no effects of housing condition or genotype on expression of NR3C2 [Panel **(E)**]. * *p* < 0.05 for difference between genotypes; & *p* < 0.05 for difference between HE and LE; # *p <* 0.05 for difference between males and females. For the number of rats per group, see Table 1.

Expression of the stress response transcription factor NRF2 was affected by housing condition, but this effect was independently interacting with sex and with genotype (Figure 7A). Thus, in addition to a main effect of housing condition [*F*(1,80) = 8.02, *p* = 0.006, *η_p_^2^* = 0.091], ANOVA revealed both a main effect of sex [*F*(1,80) = 30.5, *p* < 0.001, *η_p_^2^* = 0.276] and a sex × housing interaction [*F*(1,80) = 7.62, *p* = 0.007, *η_p_^2^* = 0.087] as well as a main effect of genotype [*F*(2,80) = 3.68, *p* = 0.029, *η_p_^2^* = 0.084] and a genotype × housing interaction [*F*(1,80) = 4.03, *p* = 0.022, *η_p_^2^* = 0.092], but no sex × genotype × housing condition interaction. Further analysis, split by sex of the animals, revealed significantly lower NRF2 expression in females in the HE condition compared to the LE condition, independent of genotype [*F*(1,42) = 12.40, *p* = 0.001, *η_p_^2^* = 0.228], but no housing condition effect in males (Figure 7A). Analysis of the data split by housing condition showed a main effect of sex [*F*(1,40) = 39.4, *p* < 0.001, *η_p_^2^* = 0.496], with NRF2 expression in the LE condition higher in females than in males (Figure 7A). There was also a main effect of genotype in the LE condition [*F*(2,40) = 8.64, *p* < 0.001, *η_p_^2^* = 0.302] with NRF2 expression higher in Met/Met rats compared to Val/Val [*F*(1,24) = 4.47, *p* = 0.045, *η_p_^2^* = 0.157] and Val/Met rats [*F*(1,28) = 18.2, *p* < 0.001, *η_p_^2^* = 0.394] but not between Val/Val and Val/Met rats (Figure 7A). There were no differences between the groups in the HE condition (Figure 7A).

There were no effects of genotype or housing condition and no sex differences in the expression of SGK1 (Figure 7B) or FKBP5 (Figure 7C). Expression of the glucocorticoid receptor gene, NR3C1 (Figure 7D), was significantly lower in females than in males [*F*(1,85) = 14.6, *p* < 0.001, *η_p_^2^* = 0.146], and there was also a genotype × housing condition × sex interaction [*F*(2,85) = 6.16, *p* = 0.003, *η_p_^2^* = 0.127]. In males, there was only a genotype effect [*F*(2,42) = 4.27, *p* = 0.020, *η_p_^2^* = 0.169]. Both Val/Met [*F*(1,32) = 4.73, *p* = 0.037, *η_p_^2^* = 0.129] and Met/Met males [*F*(1,26) = 6.97, *p* = 0.014, *η_p_^2^* = 0.211] showed lower NR3C1 expression than Val/Val males, with no differences between Val/Met and Met/Met males (Figure 7D). In females, there was a genotype × housing condition interaction [*F*(2,43) = 6.30, *p* = 0.004, *η_p_^2^* = 0.227]. While there were no genotype differences in females in either the LE condition or the HE condition, analysis of the data split by genotype revealed that NR3C1 expression was higher in Met/Met females in the HE condition than in the LE condition [*F*(1,15) = 13.65, *p* = 0.002, *η_p_^2^* = 0.476] with no housing condition effects in either Val/Val or Val/Met females (Figure 7D).

There were no effects of housing condition or genotype on expression of the mineralocorticoid receptor, NR3C2 (Figure 7E).

## Discussion

The aim of this study was to investigate the effect of early-life environment on anxiety-like behavior in adulthood and the modulating role of BDNF Val66Met. We compared adult rats from a prenatal/neonatal high-enrichment/high-complexity environment (HE) with those from a low-enrichment/low-complexity environment (LE). The main results indicated significant effects of early-life environment, with some effects occurring in both males and females, but other effects occurring only in females (Table 2). Specifically, body weight was higher in both males and females of HE rats compared to LE rats. Anxiety-like behavior, expressed as time on the open arms of the plus maze or time in the inner zone of the open field, was greater in both male and female HE rats compared to LE rats. In contrast, adrenal weight was increased only in female HE rats, distance travelled on the plus maze was decreased only in female HE rats, and forced swim test immobility was reduced, and swimming was increased, by HE only in females. Fear conditioning and fear extinction, other indices of anxiety-like behavior, were notably not altered by early-life environmental conditions.

**Table 2:** Summary of the results comparing HE with LE.

Parameter	Comparing HE vs. LE	Figure
Body weight	Higher in both males and females	Figure 1
Adrenal weight	Higher in females	Figure 2
Plus maze open arm time	Lower in males and females	Figure 3A
Plus maze distance moved	Lower in females	Figure 3B
Open field inner zone time	Lower in males and females	Figure 3C
Open field distance moved	No difference	Figure 3D
Fear conditioning	No difference	Figure 4
Forced swim test immobility time	Lower in female Met/Met	Figure 5A
Forced swim test climbing time	Higher in females	Figure 5B
Ventral hippocampus gene expression		
BDNF	Lower in females, mostly Val/Met	Figure 6A
BDNF IV	No differences	Figure 6B
BDNF 3’UTR Long	Higher in male Val/Val	Figure 6C
BDNF VI	No difference	Figure 6D
BDNF IX	No difference	Figure 6E
NRF2	Lower in females, mostly Met/Met	Figure 7A
SGK1	No difference	Figure 7B
FKBP5	No difference	Figure 7C
NR3C1	Higher in female Met/Met	Figure 7D
NR3C2	No difference	Figure 7E

A few of the behavioral changes, depending on early-life environment, were dependent on BDNF genotype. In contrast, the effect of HE vs. LE condition on expression of BDNF, NRF2, and the glucocorticoid receptor, NR3C1, in the ventral hippocampus varied depending on genotype, and most of these changes were again only seen in females (Table 2). Thus, BDNF expression was lower in HE females than LE females, with the greatest effect seen in Val/Met rats, with no such effect in males. Expression of NRF2 was also decreased in females, with the greatest change seen in Met/Met rats. Finally, compared to the LE condition, expression of NR3C1 was higher in HE female Met/Met rats only. Of note, there were no effects of early-life environment or genotype on the expression of SGK1, FKBP5, and the mineralocorticoid receptor, NR3C2.

The present study is novel in several aspects. First, while previous studies have investigated the effects of prenatal (Buss et al., 2012; Creutzberg et al., 2021; Weinstock, 2017) or neonatal stress (Gobinath et al., 2014; Millstein and Holmes, 2007; Nishi, 2020) on anxiety-like behavior in rats, few of these studies have focused on housing conditions as a form of prenatal/neonatal stress during early development, with the offspring being housed in identical conditions after weaning. Our protocol thus focuses on the long-lasting developmental effects of early-life environmental conditions rather than continued exposure to these conditions. Second, while the effect of different cage types (Logge et al., 2013; Shan et al., 2014) and environmental enrichment (Dominguez-Oliva et al., 2025; Huttenrauch et al., 2016; Singhal et al., 2019) on anxiety-like behaviors has been studied previously, those studies mostly focused on the effect of these conditions in already adolescent or adult rats, not the long-term effect of these conditions during the prenatal/neonatal period. Third, while it is widely accepted that prenatal/neonatal stress, as well as environmental enrichment, affects BDNF signaling in the brain (Notaras and van den Buuse, 2020; Roceri et al., 2004), no previous studies have addressed the interaction of the common human BDNF gene variant, Val66Met, with the effects of prenatal/neonatal environmental factors. Finally, few studies have addressed the long-term effects of early housing conditions on molecular aspects of BDNF gene expression or on the expression of specific stress markers in the ventral hippocampus.

### Long-term effects of prenatal/neonatal housing conditions on anxiety-like behavior

Body weight was significantly higher in both male and female HE than LE offspring, independent of genotype. Several studies have shown that the pre- and perinatal environment can have enduring effects on the body weight development of offspring (Burgueno et al., 2020; Tamashiro and Moran, 2010). Further experiments should be done to identify the metabolic and endocrine factors involved in the differences in body weight between the groups. Because the focus of this study was the behavioral and molecular consequences of early developmental factors, these factors were not studied here. However, it is important to note that the subtle body weight differences are unlikely to explain any of the behavioral changes seen between the LE and HE conditions.

### Long-term effects of prenatal/neonatal housing conditions on anxiety-like behavior

It was hypothesized that rats in HE conditions would present lower indices of anxiety-like behaviors than rats in LE conditions, similar to the protective effects of environmental enrichment on the effects of prenatal/neonatal stress seen in previous studies (Koe et al., 2016; Rule et al., 2021). It was also predicted that Met/Met rats would present the highest rate of anxiety-like behaviors due to their reduced activity-dependent BDNF release in comparison to Val/Met and Val/Val genotypes (Chen et al., 2006). However, behavioral analysis revealed that rats reared in HE conditions spent less time in the open arms of the EPM and less time in the inner zone of the open field than those from LE conditions, suggesting a high-anxiety phenotype independent of Val66Met genotype. There has been some controversy regarding the term “environmental enrichment” to indicate several environmental factors such as group housing, larger cage size, and addition and regular changing of environmental items such as toys, ladders, and nesting material (Ratuski and Weary, 2022; Lopes et al., 2018; Toth et al., 2011). Such conditions have, in fact, been shown to result in profound changes in hypothalamic–pituitary–adrenal axis function consistent with moderate stress levels (Konkle et al., 2010; Moncek et al., 2004). Thus, it is possible that our HE protocol had a similar impact to moderate prenatal/neonatal stress rather than a low-stress environment, which could explain our finding of lasting higher indices of high-anxiety behavior in adulthood. Indeed, several studies have shown profound long-lasting changes in HPA axis activity and anxiety-like behavior in adulthood following prenatal stress (Creutzberg et al., 2021; Weinstock, 2017; Abe et al., 2007; Maccari and Morley-Fletcher, 2007). Specifically in our study, during the neonatal period, the frequent changes in the environment could have caused mild/moderate stress for the dam and offspring, with long-term effects in both male and female rats (Ennaceur et al., 2006; Tang et al., 2006). We generally did not observe any differences between Val/Val, Val/Met, and Met/Met rats in the long-lasting effects of early-life environment on anxiety-like behavior. Thus, it may be that the effects of early life environment in this rat model override any modulatory effect of the Val66Met genotype. This is in contrast to previous studies using Val66Met mice, where Met/Met mice showed higher levels of anxiety-like behavior (Chen et al., 2006) and greater sensitivity to stress (Vandenberg et al., 2018).

While our model showed significant differences in anxiety-like behavior on the plus-maze and in the open field, there were no changes in fear conditioning, another behavioral paradigm associated with fear and anxiety. Previous studies have shown a similar dissociation, suggesting that, at least in animal models, general anxiety, such as caused by innate aversion of open spaces in rats and mice, is regulated differently from conditioned fear and controlled by different neural pathways (Daniel-Watanabe and Fletcher, 2022; Heinz et al., 2021; Perusini and Fanselow, 2015). Our results suggest that neural pathways involved in general anxiety, such as the Bed Nucleus of the Stria Terminalis and its projections, are more sensitive to stressful early-life housing conditions than those mediating learned fear, such as the amygdala (Perusini and Fanselow, 2015; Pego et al., 2008), although further studies are needed to confirm this.

### Additional differences between HE and LE conditions

In addition to the complexity and frequent changes in the environment, there are several other factors that could have played a role in the long-term effects of HE vs. LE on anxiety-like behavior. For example, it was routinely observed that HE dams would spend considerable time on the ‘mezzanine’ platform of their multilevel cage, that is, away from the litter, whereas in the absence of such a platform, the LE dams would tend to remain in the nest with their offspring, potentially leading to differences in the volume of maternal care. Although future experiments should include quantitative analysis of maternal behavior to confirm these observations, it should be noted that maternal separation is a well-known method of neonatal stress, leading to enhanced anxiety-like behavior in adulthood (Wang et al., 2020). Similarly, reduced maternal care may lead to increased anxiety-like behavior in the offspring (Caldji et al., 1998; Liu et al., 1997), similar to what we observed here.

It should furthermore be noted that the open-top cages we used in the LE condition here allow more social communication between cages, in the form of pheromone scent and ultrasonic vocalizations, than the closed IVC cages with their isolated ventilation (Bigelow et al., 2022; Carew and Ghosh, 2020), potentially offsetting some of the effects of other cage-related factors such as reduced cage height, causing reduced pandiculation and less nesting material, causing less burrowing (Makowska and Weary, 2016). Future experiments can aim to limit the number of differences between our HE and LE conditions, for example, by housing LE rats in the larger IVC cages but without added enrichment.

Finally, as part of the ‘enrichment’ in the HE condition, the rats were provided with sunflower seeds to stimulate foraging behavior. Given the high fat and high protein content of these seeds, this could have constituted dietary supplementation and may have played a role in the significantly higher body weight in HE rats than LE rats. Given that these seeds are rich in phytoestrogens, their supplementation could have also played a role in the sex differences seen in some of the behavioral outcomes of the HE vs. LE conditions. However, it should be noted that no sunflower seeds were provided in the ‘standard’ housing, where all rats were kept post-weaning.

### Sex differences in the long-term effects of prenatal/neonatal housing conditions

While some of the behavioral changes in adulthood following prenatal/neonatal HE were seen in both males and females, other effects were only seen in females, and, notably, there were no behavioral effects that were only present in male offspring. For example, female HE showed reduced plus maze overall distance moved, reduced forced swim test immobility, and increased swimming time. Similarly, female HE rats showed enlarged adrenals, consistent with hyperactivity of the HPA axis (Ulrich-Lai et al., 2006). This could indicate that females show a greater sensitivity to the effects of early-life environment than males, as has been suggested by others (Weinstock, 2007) and consistent with the observation of a higher prevalence of anxiety-like conditions in women than in men (Altemus et al., 2014; Gater et al., 1998). However, previous studies on sex differences in the effects of prenatal or neonatal stress have found varying results, likely caused by differences in the type and timing of the stress (Creutzberg et al., 2021; Gobinath et al., 2014). For example, repeated neonatal separation caused greater increases in anxiety-like behavior in male than in female offspring (de Melo et al., 2018) although there were no sex differences in other studies (Kalinichev et al., 2002; Soares-Cunha et al., 2018) similar to our findings. On the other hand, more specifically about forced swim stress, our findings are similar to previous studies showing greater effects of prenatal or neonatal stress in female than male offspring (Alonso et al., 1991; Goodwill et al., 2019).

### Long-term effects of prenatal/neonatal housing conditions on BDNF expression

Similar to some of the behavioral changes between HE and LE observed only in female offspring, we observed a significantly lower BDNF expression in the ventral hippocampus in female, but not male rats, with the greatest effect seen in Val/Met rats. BDNF expression is under the control of several promoters, allowing complex regulatory mechanisms and resulting in many isoforms of BDNF mRNA, while all are translated into one BDNF protein (Notaras and van den Buuse, 2019; Aid et al., 2007). Of these isoforms, BDNF IV is known to be heavily regulated by neuronal activity (Zheng et al., 2012), and up-regulation of BDNF IV has previously been shown to result in attenuated contextual fear expression (Bach et al., 2023). However, in our study, there were no differences in BDNF IV levels between the groups in the ventral hippocampus, and there were also no changes in fear acquisition or extinction. BDNF 3’UTR Long is important for BDNF expression in dendrites vs. soma (Zheng et al., 2012) and is down-regulated by chronic stress (Oh et al., 2019). Our results show that BDNF 3’UTR Long expression was higher in HE Val/Val males than in LE Val/Val males, but there were no other differences in males or in females, which could explain the reduced BDNF expression or changes in anxiety-like behavior. Therefore, the changes in BDNF expression in the ventral hippocampus appear to be mediated by effects of HE vs. LE on promoters other than BDNF IV or BDNF 3’UTR Long. The extended duration of the changes in BDNF expression, several weeks following transfer of the rats from either HE or LE into a standardized environment, furthermore suggests lasting epigenetic alterations of these as yet to be identified alternative regulatory pathways (Zheng et al., 2012).

### Long-term effects of prenatal/neonatal housing conditions on stress marker expression in the ventral hippocampus

There were differential effects of housing condition and BDNF genotype on stress marker expression in the ventral hippocampus, although none of these directly paralleled the changes in behavior observed. In the LE condition, NRF2 expression was higher in male and female Met/Met rats compared to Val/Val and Val/Met rats. In addition to being a transcription factor regulating cellular responses against toxic and oxidative stress (He et al., 2020), NRF2 has been shown to stimulate BDNF expression (Yao et al., 2021), and the higher expression of NRF2 in Met/Met rats may be a compensatory response to reduced activity-dependent BDNF release in this genotype. However, others have argued that the role of NRF2 in anxiety and depression is independent of BDNF (Mendez-David et al., 2015). Indeed, there were no differences between the genotypes in the HE condition, suggesting that stress can affect NRF2 expression independent of the Val66Met genotype. Female, but not male HE rats, furthermore showed decreased NRF2 expression, independent of genotype, consistent with the effect of chronic stress and elevated corticosterone levels causing reduced NRF2 expression in previous studies (Yao et al., 2021). These data show that expression of NRF2 was regulated in a complex manner by genotype, sex, and early-life housing condition, consistent with previous studies (Bhandari et al., 2021; He et al., 2020).

SGK1 and FKBP5 have been implicated in cellular stress responses, and in previous studies, the expression of these markers was increased by early-life stress (Millette et al., 2025; Criado-Marrero et al., 2019) and in depression (Dattilo et al., 2020). However, there were no significant differences between HE and LE rats in the expression levels of these two markers, illustrating the specificity of the effect of early-life housing conditions on some aspects of anxiety-like behavior and their underlying molecular mechanisms.

Finally, the effect of stress-induced elevated HPA axis activity involves two types of glucocorticoid receptors in the brain: the glucocorticoid receptor, encoded by the nuclear receptor subfamily 3 group C member 1 (NR3C1) gene, and the mineralocorticoid receptor, encoded by the NR3C2 gene (Mifsud and Reul, 2018). Early-life stress has been shown to lead to long-term epigenetic changes in NR3C1 and NR3C2 expression (Palma-Gudiel et al., 2015; Siller Wilks et al., 2024). In our study, there was no effect of housing condition on NR3C1 expression in males, although both Val/Met and Met/Met males showed lower NR3C1 expression than Val/Val males. In contrast, in females, NR3C1 expression was higher in Met/Met females in the HE condition than in the LE condition, with no differences in Val/Val or Val/Met females. There were no effects of housing condition or genotype on the expression of NR3C2.

### Implications for housing and husbandry of experimental animals in research

It should be mentioned that, while the main focus of this study was the role of early-life environment in anxiety disorders and the modulating role of BDNF Val66Met, our findings may have importance for our understanding of the long-term effects of housing conditions of experimental animals in research. Our results suggest that the addition of extensive ‘enrichment’ may lead to enhanced anxiety-like behavior in offspring. Thus, for appropriate evaluation of study results, at the very least, it is highly important that housing condition details are reported, including cage size, type of enrichment and frequency of its being changed, food, ventilation, and possibility for inter-cage communication. As mentioned above, previous studies have highlighted differences in behavior, HPA activity, and gene expression between cage types and the effects of environmental enrichment (Dominguez-Oliva et al., 2025; Huttenrauch et al., 2016; Logge et al., 2013; Shan et al., 2014; Singhal et al., 2019). Our study shows that these factors can have even very early and life-long effects on behavior and brain gene expression, with potential ramifications for experimental results.

## Conclusion

Changes in prenatal and neonatal environmental factors may result in persistent changes in anxiety-like behavior and gene expression of BDNF and selected stress markers in adulthood, with some of these effects showing strict sex- and Val66Met genotype specificity. These results may be important for our understanding of the role of stress and the developmental environment in clinical anxiety and depression, and may also have implications for our understanding of the effects of animal housing factors in the laboratory setting.

## Data Availability

The original contributions presented in the study are publicly available. This data can be found here: https://doi.org/10.6084/m9.figshare.32754111.
