# Correction: Prenatal and neonatal housing conditions affect anxiety-like behavior in adulthood in rats and interact with brain-derived neurotrophic factor (BDNF) Val66Met to alter expression of BDNF and stress markers in the ventral hippocampus

**DOI:** 10.3389/fnmol.2026.1992919

**Published:** 2026-09-28

**Authors:** Maarten van den Buuse, Michelle Corrone, Emily J. Jaehne, Veronica Begni, Alessia Marchesin, Marco A. Riva

**Affiliations:** 1 School of Psychology and Public Health, La Trobe University, Melbourne, VIC, Australia; 2 Biological Psychiatry Unit, IRCCS Istituto Centro San Giovanni di Dio Fatebenefratelli, Brescia, Italy; 3 Department of Pharmacological and Biomolecular Sciences, University of Milan, Milan, Italy

**Keywords:** brain-derived neurotrophic factor, neonatal, prenatal, sex differences, stress

In the published article, in the **Abstract**, the headings were erroneously included in incorrect positions. The Abstract should now be corrected to:

**Introduction:** We investigated the interaction of the brain-derived neurotrophic factor (BDNF) gene variant, Val66Met, with the effect of prenatal/neonatal environmental conditions on anxiety-like behavior in adulthood in rats.

**Methods:** In a genetic Val66Met rat model, we compared the effects of a high-enrichment/high-complexity early-life environment (HE) and a low-enrichment/low-complexity environment (LE).

**Results:** Body weight was higher in both male and female HE rats compared to LE rats. Anxiety-like behavior on a plus maze or in an open field was enhanced in both male and female HE rats compared to LE rats. In contrast, following HE, only in females, adrenal weight was higher, and in the forced swim test, immobility was lower, and swimming was higher. Body weight and behavioral changes did not differ between BDNF genotypes. Fear conditioning and extinction were not affected. The effect of HE vs. LE condition on expression of BDNF, the antioxidant transcription factor, NRF2, and the glucocorticoid receptor, NR3C1, in the ventral hippocampus varied depending on genotype, and most of these changes were again only seen in females. There were no effects on the expression of the stress markers, SGK1 and FKBP5, or the mineralocorticoid receptor, NR3C2.

**Discussion:** These results show persistent effects of early-life environment on anxiety-like behavior and gene expression of BDNF and stress markers in adulthood, with some effects showing sex- and Val66Met genotype specificity. These results may be important for our understanding of factors involved in the development of clinical anxiety and depression, and also have implications for animal welfare in the laboratory setting.

The original version of this article has been updated.

