# Supplementary material for: Prenatal and neonatal housing conditions affect anxiety-like behavior in adulthood in rats and interact with brain-derived neurotrophic factor (BDNF) Val66Met to alter expression of BDNF and stress markers in the ventral hippocampus
Source: Front Mol Neurosci. 2026 Jul 8;19:1844602. doi: 10.3389/fnmol.2026.1844602 (PMC13388326; doi:10.3389/fnmol.2026.1844602)
Supplement: Supplementary file 1 [file Data_Sheet_1.DOCX]

- Supplementary Tables –

**Prenatal and neonatal housing conditions affect anxiety-like behaviour in adulthood in rats and interact with Brain-Derived Neurotrophic Factor (BDNF) Val66Met to alter expression of BDNF and stress markers in the ventral hippocampus**

Maarten van den Buuse^1,^*, Michelle Corrone^1^, Emily J. Jaehne^1^, Veronica Begni^2^, Alessia Marchesin^3^ & Marco A. Riva^2,3^

(1) School of Psychology and Public Health, La Trobe University, Melbourne, Australia

(2) Biological Psychiatry Unit, IRCCS Istituto Centro San Giovanni di Dio Fatebenefratelli, Brescia, Italy

(3) Department of Pharmacological and Biomolecular Sciences, University of Milan, Milan, Italy

**Supplementary Figure 1**

Timeline of the study. Following mating and until weaning of the offspring, rats were kept either in high enrichment/high complexity environment (HE, panels A and B) or a low enrichment/low complexity environment (LE, panel C). From weaning, the offspring was kept in standard IVC conditions with low/moderate enrichment (panel D). Behavioural testing was done from 8-11 weeks of age.

**
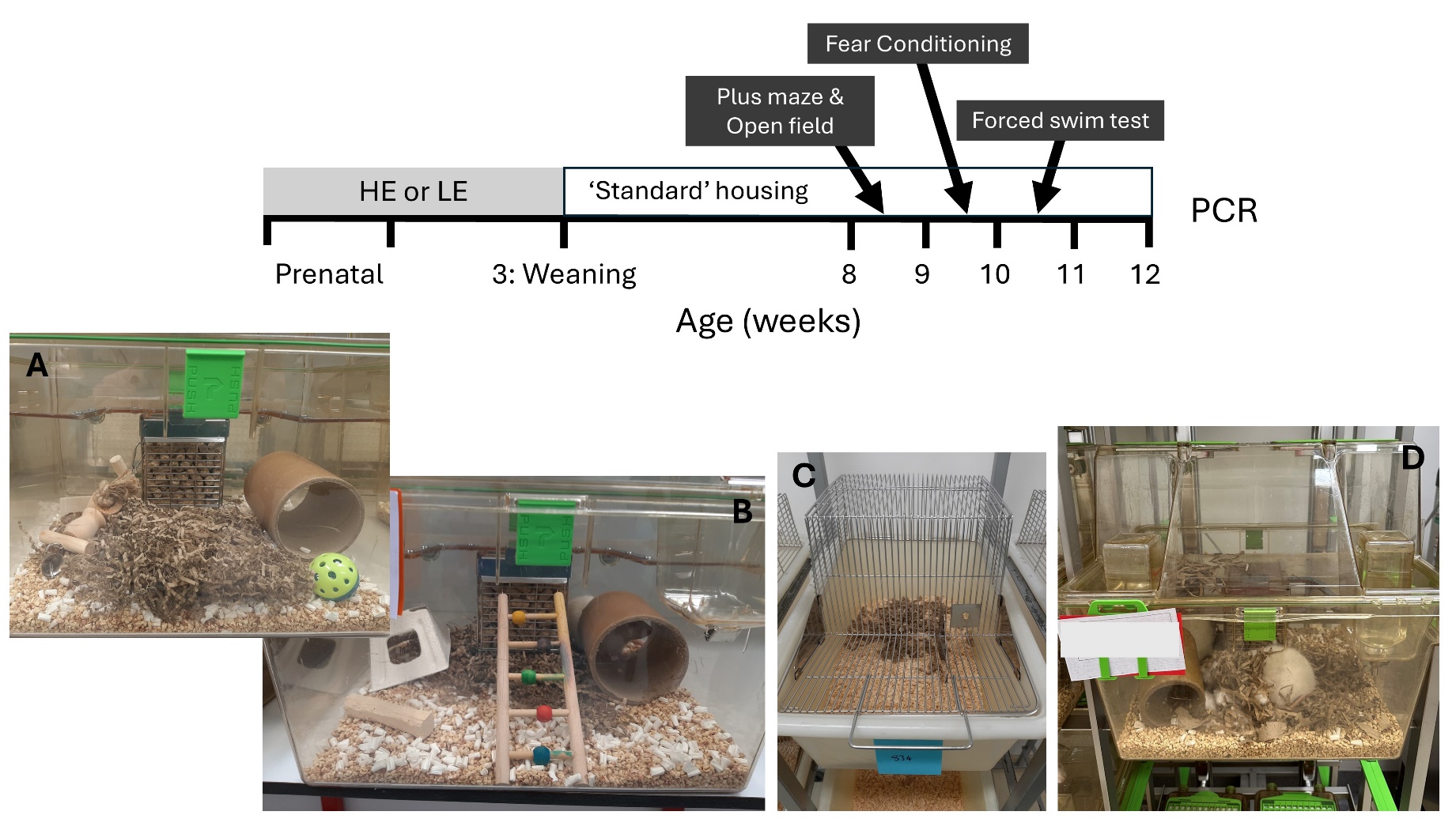
Supplementary Table 1:** *Dam age, litter size, genotype distribution, male/female ratios and pup age at weaning for all litters.*

|  | Dam age at mating (w) | Litter size (n) |  | Offspring genotype (n) | | |  | Offspring sex (n) | |  | Pup age at weaning (d) |
| --- | --- | --- | --- | --- | --- | --- | --- | --- | --- | --- | --- |
|  |  |  |  | Val/Val | Val/Met | Met/Met |  | Male | Female |  |  |
| All dams (28) | 9.4 ± 0.2 | 13.2 ± 0.5 |  | 3.2 ± 0.3 | 6.8 ± 0.5 | 3.2 ± 0.3 |  | 6.8 ± 0.3 | 6.4 ± 0.4 |  | 21.4 ± 0.2 |
| LE dams (14) | 9.6 ± 0.3 | 13.1 ± 0.7 |  | 3.4 ± 0.5 | 6.2 ± 0.8 | 3.5 ± 0.5 |  | 6.9 ± 0.5 | 6.2 ± 0.4 |  | 21.6 ± 0.3 |
| HE dams (14) | 9.3 ± 0.3 | 13.3 ± 0.8 |  | 3.1 ± 0.3 | 7.3 ± 0.6 | 2.9 ± 0.4 |  | 6.8 ± 0.4 | 6.5 ± 0.8 |  | 21.1 ± 0.2 |

Data are mean ± SEM.

**Supplementary Table 2:** *Litter size from which selected experimental groups were obtained by sex, genotype and enrichment condition.*

|  | Males (n) | | | | |  | Females (n) | | | | |
| --- | --- | --- | --- | --- | --- | --- | --- | --- | --- | --- | --- |
| Genotype/  Condition | Val/Val |  | Val/Met |  | Met/Met |  | Val/Val |  | Val/Met |  | Met/Met |
| LE | 14.1 ± 0.8 |  | 13.3 ± 0.8 |  | 12.4 ± 0.8 |  | 13.7 ± 0.9 |  | 13.2 ± 0.8 |  | 14.3 ± 0.7 |
| HE | 14.0 ± 1.1 |  | 13.4 ± 0.9 |  | 12.9 ± 0.9 |  | 13.1 ± 1.3 |  | 13.8 ± 0.9 |  | 14.8 ± 0.8 |

Data are mean ± SEM.

**Supplementary Table 3:** Plus maze distance moved, zone visit time, and number of arm visits.

| Arm | | Total distance (cm) |  | Zone visit time (sec) | | | |  | Number of arm entries | | |
| --- | --- | --- | --- | --- | --- | --- | --- | --- | --- | --- | --- |
|  |  |  |  | Open arms | Closed arms | Centre zone | Closed + Centre |  | Open | Closed | Total |
| Male | Val/Val LE | 1365 ± 78 |  | 28.3 ± 7.6 | 181.9 ± 13.6 | 89.8 ± 9.1 | 271.7 ± 7.6 |  | 4.4 ± 1.1 | 12.5 ± 0.9 | 16.9 ± 1.5 |
|  | Val/Val HE | 1413 ± 78 |  | 22.6 ± 7.5* | 183.2 ± 8.6 | 94.2 ± 6.4 | 277.4 ± 7.5* |  | 4.0 ± 1.0 | 11.9 ± 0.8 | 15.9 ± 1.4 |
|  | Val/Met LE | 1196 ± 56 |  | 21.6 ± 6.2 | 210.0 ± 9.5 | 68.5 ± 5.7 | 278.4 ± 6.2 |  | 3.2 ± 0.6 | 10.7 ± 0.9 | 13.9 ± 1.1 |
|  | Val/Met HE | 1341 ± 85 |  | 16.4 ± 6.1* | 193.2 ± 12.6 | 90.4 ± 8.4 | 283. 6 ± 6.1* |  | 4.6 ± 1.2 | 13.2 ± 1.4 | 17.8 ± 1.8 |
|  | Met/Met LE | 1241 ± 93 |  | 21.2 ± 5.2 | 190.2 ± 10.6 | 88.6 ± 7.0 | 278.8 ± 5.2 |  | 4.7 ± 1.3 | 10.2 ± 0.8 | 14.9 ± 1.6 |
|  | Met/Met HE | 1242 ± 106 |  | 8.4 ± 4.6* | 205.5 ± 12.1 | 86.2 ± 9.7 | 291.6 ± 4.6* |  | 2.7 ± 1.0 | 12.3 ± 1.3 | 15.0 ± 1.9 |
| Female | Val/Val LE | 1570 ± 107 |  | 44.3 ± 11.5 | 175.9 ± 11.7 | 79.8 ± 5.6 | 255.7 ± 11.5 |  | 7.2 ± 1.5 | 12.7 ± 1.3 | 19.9 ± 2.3 |
|  | Val/Val HE | 1285 ± 64* |  | 10.7 ± 4.2* | 202.7 ± 8.2 | 86.7 ± 5.2 | 289.2 ± 4.2* |  | 3.3 ± 0.9 | 12.4 ± 1.1 | 15.6 ± 1.5 |
|  | Val/Met LE | 1547 ± 60 |  | 38.2 ± 7.5 | 176.4 ± 10.6 | 85.5 ± 3.6 | 261.8 ± 7.5 |  | 7.5 ± 1.4 | 13.7 ± 1.0 | 21.3 ± 1.7 |
|  | Val/Met HE | 1340 ± 94* |  | 26.5 ± 4.8* | 195.0 ± 8.9 | 78.5 ± 5.6 | 273.5 ± 4.8* |  | 5.3 ± 0.9 | 11.8 ± 1.2 | 17.0 ± 1.7 |
|  | Met/Met LE | 1463 ± 85 |  | 42.7 ± 7.7 | 178.5 ± 11.2 | 78.8 ± 6.6 | 257.3 ± 7.7 |  | 5.4 ± 1.1 | 12.7 ± 1.7 | 18.1 ± 1.9 |
|  | Met/Met HE | 1439 ± 77* |  | 31.3 ± 7.6* | 185.9 ± 12.2 | 82.8 ± 6.8 | 268.7 ± 7.6* |  | 5.9 ± 1.2 | 14.1 ± 1.2 | 20.0 ± 1.7 |
| All | LE | 1396 ± 36 |  | 32.6 ± 3.3 | 185.7 ± 4.6 | 81.7 ± 2.7 | 267.4 ± 3.3 |  | 5.4 ± 0.5 | 12.1 ± 0.5 | 17.5 ± 0.7 |
|  | HE | 1350 ± 35 |  | 20.1 ± 2.6* | 193.3 ± 2.9 | 86.5 ± 2.9 | 279.9 ± 2.6* |  | 4.4 ± 0.4 | 12.6 ± 0.5 | 17.0 ± 0.7 |

Data are mean ± SEM. * *p* <0.05 for difference between LE and HE condition based on main ANOVA effect.

**Supplementary Table 4:** Open field distance moved, zone visit time, and number of arm visits.

|  | | Total distance (cm) |  | Zone visit time (sec) | |  | Number of zone entries | |
| --- | --- | --- | --- | --- | --- | --- | --- | --- |
|  |  |  |  | Inner | Outer |  | Inner | Outer |
| Male | Val/Val LE | 6474 ± 257 |  | 48.5 ± 8.9 | 548.2 ± 8.7 |  | 17.3 ± 2.1 | 22.4 ± 2.9 |
|  | Val/Val HE | 6509 ± 347 |  | 37.0 ± 8.6* | 561.2 ± 8.6 |  | 15.7 ± 3.7 | 20.1 ± 3.6 |
|  | Val/Met LE | 5382 ± 325 |  | 32.7 ± 8.9 | 564.0 ± 8.8 |  | 12.9 ± 3.2 | 16.8 ± 3.0 |
|  | Val/Met HE | 6437 ± 351 |  | 35.4 ± 9.7* | 562.7 ± 9.9 |  | 11.8 ± 2.8 | 18.3 ± 3.1 |
|  | Met/Met LE | 5774 ± 401 |  | 43.9 ± 10.7 | 552.6 ± 11.4 |  | 13.2 ± 3.3 | 21.9 ± 6.1 |
|  | Met/Met HE | 5802 ± 382 |  | 16.2 ± 5.0* | 578.9 ± 5.3 |  | 7.6 ± 2.2 | 16.7 ± 3.9 |
| Female | Val/Val LE** | 7346 ± 403 |  | 52.5 ± 11.5 | 539.2 ± 10.8 |  | 20.8 ± 3.7 | 40.3 ± 3.5 |
|  | Val/Val HE | 7010 ± 334 |  | 51.4 ± 10.7* | 536.2 ± 11.1 |  | 17.5 ± 2.1 | 37.5 ± 6.7 |
|  | Val/Met LE** | 7571 ± 360 |  | 56.3 ± 10.2 | 532.7 ± 11.3 |  | 23.3 ± 3.6 | 40.5 ± 6.2 |
|  | Val/Met HE | 6621 ± 319 |  | 45.3 ± 7.2* | 545.2 ± 7.7 |  | 18.3 ± 2.3 | 38.4 ± 5.0 |
|  | Met/Met LE** | 6991 ± 320 |  | 66.1 ± 7.3 | 527.1 ± 7.6 |  | 21.4 ± 2.3 | 37.5 ± 5.4 |
|  | Met/Met HE | 6712 ± 225 |  | 41.8 ± 7.7* | 546.0 ± 7.9 |  | 19.0 ± 2.4 | 37.9 ± 6.3 |
| All | LE | 6582 ± 169 |  | 49.6 ± 3.9 | 544.4 ± 4.1 |  | 18.0 ± 1.3 | 29.7 ± 2.2 |
|  | HE | 6532 ± 138 |  | 38.6 ± 3.6* | 554.6 ± 3.9 |  | 15.2 ± 1.2 | 28.1 ± 2.3 |

Data are mean ± SEM. * *p* <0.05 for difference between LE and HE condition based on main ANOVA effect. ** *p* < 0.05 for difference between LE male and LE female rats.

**Supplementary Table 5:** Average %freezing during fear conditioning acquisition, fear memory and extinction learning testing.

*

|  | | Day 1  CS1-3 |  | Day 2: Extinction learning | |  |
| --- | --- | --- | --- | --- | --- | --- |
|  |  |  |  | CS1-10 | CS1-40 |  |
| Male | Val/Val LE | 33.9 ± 8.2  * |  | 72.3 ± 6.8  * | 27.0 ± 4.1  * |  |
|  | Val/Val HE | 34.1 ± 5.5 |  | 78.1 ± 5.0 | 31.0 ± 4.0 |  |
|  | Val/Met LE | 42.1 ± 6.5 |  | 77.8 ± 6.6 | 47.8 ± 7.4 |  |
|  | Val/Met HE | 40.1 ± 5.8 |  | 84.3 ± 6.7 | 38.2 ± 4.4 |  |
|  | Met/Met LE | 48.4 ± 5.5 |  | 81.4 ± 8.3 | 41.4 ± 8.3 |  |
|  | Met/Met HE | 40.6 ± 8.8 |  | 82.0 ± 10.8 | 37.5 ± 7.4 |  |
| Female | Val/Val LE | 31.0 ± 5.5 |  | 66.3 ± 6.7 | 22.2 ± 4.1 |  |
|  | Val/Val HE | 44.3 ± 4.2 |  | 80.3 ± 5.0 | 28.6 ± 4.1 |  |
|  | Val/Met LE | 27.0 ± 4.7 |  | 58.6 ± 8.7 | 23.9 ± 4.9 |  |
|  | Val/Met HE | 34.0 ± 6.5 |  | 63.7 ± 9.0 | 21.5 ± 3.4 |  |
|  | Met/Met LE | 30.0 ± 6.5 |  | 55.7 ± 12.5 | 21.6 ± 5.5 |  |
|  | Met/Met HE | 31.8 ± 7.5 |  | 59.2 ± 10.5 | 19.9 ± 3.7 |  |
| All | LE | 35.4 ± 2.6 |  | 68.7 ± 3.5 | 30.8 ± 2.7 |  |
|  | HE | 37.1 ± 2.6 |  | 74.3 ± 3.4 | 29.3 ± 2.0 |  |

Data are mean ± SEM. * *p* < 0.05 for difference between male and female rats.

**Supplementary Table 6:** List of probes and primers used in gene expression analysis.

| Gene | | | Applera code |
| --- | --- | --- | --- |
| β-ACT | | | rn00667869_m1 |
| GAPDH | | | rn99999916_s1 |
| BDNF IV | | | rn01484927_m1 |
| BDNF 3'UTR LONG | | | rn02531967_s1 |
| SGK1 | | | rn00570285_m1 |
| FKBP5 | | | rn01768371_m1 |
| NR3C1 | | | rn00561369_m1 |
| NR3C2 | | | rn00565562_m1 |
|  | | |  |
|  | | | Primer sequence |
| BDNF | FWD | AAGTCTGCATTACATTCCTCGA | |
|  | REV | GTTTTCTGAAAGAGGGACAGTTTAT | |
|  | PROBE | TGTGGTTTGTTGCCGTTGCCAAG | |
| NRF2 | FWD | ATATTCCCAGCCACGTTGAG | |
|  | REV | CCAAACTTGCTCCATGTCCT | |
|  | PROBE | GAGACGGCCATGACTGATTT | |
